# Traumatic Injury and Exposure to Mitochondrial-Derived Damage Associated Molecular Patterns Suppresses Neutrophil Extracellular Trap Formation

**DOI:** 10.3389/fimmu.2019.00685

**Published:** 2019-04-02

**Authors:** Jon Hazeldine, Robert J. Dinsdale, Paul Harrison, Janet M. Lord

**Affiliations:** ^1^Institute of Inflammation and Ageing, University of Birmingham, Birmingham, United Kingdom; ^2^National Institute for Health Research Surgical Reconstruction and Microbiology Research Centre, Queen Elizabeth Hospital Birmingham, Birmingham, United Kingdom; ^3^Scar Free Foundation Birmingham Centre for Burns Research, Queen Elizabeth Hospital Birmingham, Birmingham, United Kingdom

**Keywords:** neutrophils, neutrophil extracellular traps, trauma, mitochondrial-derived DAMPs, immune suppression

## Abstract

Major traumatic injury induces significant remodeling of the circulating neutrophil pool and loss of bactericidal function. Although a well-described phenomenon, research to date has only analyzed blood samples acquired post-hospital admission, and the mechanisms that initiate compromised neutrophil function post-injury are therefore poorly understood. Here, we analyzed pre-hospital blood samples acquired from 62 adult trauma patients (mean age 44 years, range 19–95 years) within 1 h of injury (mean time to sample 39 min, range 13–59 min). We found an immediate impairment in neutrophil extracellular trap (NET) generation in response to phorbol 12-myristate 13-acetate (PMA) stimulation, which persisted into the acute post-injury phase (4–72 h). Reduced NET generation was accompanied by reduced reactive oxygen species production, impaired activation of mitogen-activated protein kinases, and a reduction in neutrophil glucose uptake and metabolism to lactate. Pre-treating neutrophils from healthy subjects with mitochondrial-derived damage-associated molecular patterns (mtDAMPs), whose circulating levels were significantly increased in our trauma patients, reduced NET generation. This mtDAMP-induced impairment in NET formation was associated with an N-formyl peptide mediated activation of AMP-activated protein kinase (AMPK), a negative regulator of aerobic glycolysis and NET formation. Indeed, activation of AMPK via treatment with the AMP-mimetic AICAR significantly reduced neutrophil lactate production in response to PMA stimulation, a phenomenon that we also observed for neutrophils pre-treated with mtDAMPs. Furthermore, the impairment in NET generation induced by mtDAMPs was partially ameliorated by pre-treating neutrophils with the AMPK inhibitor compound C. Taken together, our data demonstrate an immediate trauma-induced impairment in neutrophil anti-microbial function and identify mtDAMP release as a potential initiator of acute post-injury neutrophil dysfunction.

## Introduction

Major injury induces significant phenotypic and functional remodeling of the peripheral neutrophil pool, attributable in part to the emergence into circulation of immature granulocytes (IGs) and highly mature neutrophil subsets (1–5). Alongside changes in the surface expression of adhesion molecules and chemokine receptors (6–8), immediate and prolonged impairments in phagocytosis (1, 9), reactive oxygen species (ROS) production (1, 3), and chemotaxis (10, 11) have been reported for neutrophils isolated from critically-injured patients. Whilst understanding of trauma-induced changes in neutrophil intracellular bactericidal function is well-developed, few studies have investigated the impact of injury on the extracellular defensive mechanisms of neutrophils and how soon after injury, any compromise occurs.

Comprised of a DNA backbone decorated with granular and cytosol-derived peptides and enzymes, neutrophil extracellular traps (NETs) are an extracellular anti-microbial defense mechanism deployed to prevent pathogen dissemination (12). Studies that have examined NET generation post-injury have reported an increase in *ex vivo* NET formation by resting neutrophils (10, 13), a hyperactivity that may reflect their *in vivo* exposure to high mobility group box-1 (HMGB-1) and interleukin (IL)-33, two NET-inducing alarmins whose circulating concentrations are significantly increased post-injury (14–16). However, in terms of stimulus-induced NET generation, comparable (13), or reduced (1, 10) NET production in response to stimulation with phorbol 12-myristate 13-acetate (PMA) has been reported post- trauma. Of these studies, only one performed quantitative analysis (1), and neither study that reported a post-injury reduction in NET formation investigated the mechanism(s) responsible (1, 10).

Activation of three non-redundant molecular processes underpin PMA-induced NET generation. Produced by the multi-subunit enzyme nicotinamide adenine dinucleotide phosphate (NADPH) oxidase, ROS generation is essential for the process of chromatin decondensation that precedes NET release (17, 18). Occuring prior to (19) or following (20) ROS production, activation of the mitogen activated protein kinases p38 and extracellular signal regulated kinase 1/2 (ERK 1/2), triggers NET formation by inhibiting caspase activation and increasing expression of the pro-survival protein Mcl-1, thus promoting NET production over the induction of apoptosis (19, 20). Finally, glycolysis is a fundamental metabolic requirement for PMA-induced NET formation, with the uptake and breakdown of extracellular glucose a necessity for the process of DNA expulsion (21).

Trauma-associated tissue damage results in the release into the circulation of damage-associated molecular patterns (DAMPs), a collection of cytosolic, mitochondrial and nuclear-derived proteins, and DNA (14, 22, 23). Whilst renowned for their role in immune activation (23, 24), data are emerging that suggests mitochondrial-derived DAMPs (mtDAMPs), which include N-formylated peptides and mitochondrial DNA (mtDNA), possess immune tolerising properties. For instance, it has been shown *in vitro* that monocytes pre-exposed to mtDNA (25) and neutrophils pre-treated with whole mtDAMP preparations (23) exhibit impaired cytokine production and calcium mobilization respectively upon secondary stimulation. Furthermore, a significant reduction in stimulus-induced ROS production and transmigration was reported for neutrophils pre-exposed to bacterial-derived or synthetic N-formylated peptides (10, 22, 26), both of which signal through the same formyl peptide receptor (FPR) as mitochondrial-derived formyl peptides. Based on these observations, the concept of mtDAMP-induced tolerance has been coined and proposed to be a potential mechanistic explanation for the state of peripheral neutrophil dysfunction that develops in the aftermath of major trauma (10, 22).

Here, in a prospective observational study of trauma patients, we have performed for the first time a quantitative assessment of NET production during the pre-hospital, ultra-early (≤60 min), and acute (4–72 h) post-injury phases, and assessed the impact that major injury has on the molecular processes and signaling pathways that underpin PMA-induced NET generation. Furthermore, based on the emerging concept of mtDAMP-induced tolerance, we have investigated whether pre-exposing neutrophils isolated from healthy subjects to mtDAMPs *in vitro* results in altered NET generation upon secondary stimulation with PMA and the mechanisms involved.

## Materials and Methods

### Study Design and Setting

This manuscript presents data acquired from subjects enrolled into the Brain Biomarkers after Trauma Study, an ongoing prospective longitudinal observational study of adult trauma patients conducted at a single Major Trauma Center site in the UK (University Hospitals Birmingham NHS Foundation Trust, Birmingham). Ethical approval for the study was granted by the North Wales Research Ethics Committee–West (REC reference: 13/WA/0399, Protocol Number: RG_13-164).

Patient enrolment began in the pre-hospital setting, where on a 24/7 basis between March 2016 and October 2018, emergency care teams acquired blood samples from adult trauma patients (≥18 years) with a suspected injury severity score (ISS) ≥8 within 1 h of injury (defined as the time of phone call to emergency services). In the pre-hospital setting, blood samples were not taken from patients who were deemed unlikely to survive transportation to hospital. Post admission, patients were excluded if they were aged <18 years, if pre-hospital blood samples had been acquired >1 h post-injury and if clinical assessments confirmed either an ISS <8 or a previous diagnosis of neuro-degenerative disease. No patients received blood products in the pre-hospital setting.

### Capacity and Consent

Due to the nature of injuries sustained, patients were unlikely to provide informed consent for their participation at the time of study enrolment. Consequently, patient recruitment was performed under the guidance of the Mental Health Capacity Act 2005 for research in emergency situations and the Declaration of Helsinki. For patients who lacked capacity, an agreement for study participation was sought from a legal consultee (family member or clinician not directly involved in the study), with written consent obtained from the patient once they regained capacity. In instances where the patient did not regain capacity, data were retained in accordance with the agreement of the legal consultee.

### Blood Sampling

In the pre-hospital environment, peripheral venous blood samples were acquired during the intravenous cannulation of patients or by venepuncture. Once taken, blood tubes were stored at room temperature (RT) until arrival at hospital, where analysis began within 1 h by a single laboratory researcher on a 24/7 basis. Additional blood samples were acquired 4–12 and 48–72 h post-injury. At all three time points, blood samples were collected into BD Vacutainers® (BD Biosciences, Oxford, UK) containing ethylenediaminetetraacetic acid, z-serum clotting activator or 1/10 volume of 3.2% trisodium citrate. Full blood counts were performed using a Sysmex XN-1000 hematology analyser (Sysmex UK, Milton Keynes, UK) that measures a white cell differential and IGs, which are defined as promyelocytes, myelocytes, and metamyelocytes. The analyser uses fluorescence dyes that label intracellular DNA and RNA, with the intensity of the fluorescence signal directly proportional to the nucleic acid content of the cell. Due to their higher RNA content, IGs are discriminated from mature neutrophils via their stronger fluorescence signal. Daily internal quality control measurements (XN check, Sysmex UK) and monthly external quality control samples (UKNEQAS, Watford, UK) ensured instrument performance.

Sixty-seven adults (mean age 31 years, range 18–80) served as a cohort of healthy controls (HCs). HCs were volunteers who were not taking any regular medication for a diagnosed illness and did not have an acute episode of infection prior to the time of sampling. The recruitment of HCs was carried out in accordance with the ethical approval granted by the University of Birmingham Research Ethics Committee (Ref: ERN_12-1184) with written informed consent from all subjects. All subjects gave written informed consent in accordance with the Declaration of Helsinki.

### Preparation of mtDAMPs and mtDNA

MtDNA and mtDAMPs were prepared from mitochondria isolated from the K562 tumor cell line (ATCC®, Teddington, Middlesex, UK) as described previously (24). MtDNA concentration and protein content within mtDAMPs were determined by spectrophotometry (Nanodrop 2000; Thermo Fisher Scientific, Paisley, UK) and preparations stored at −80°C prior to use.

### Neutrophil Isolation and Treatment

Neutrophils were isolated by Percoll density gradient centrifugation (Scientific Lab Supplies, Nottingham, UK) with cell purity, which was routinely ≥99%, determined using a Sysmex XN-1000 hematology analyser. Neutrophils were re-suspended at concentrations of 1-10 × 10^6^/ml in phenol red free or phenol red containing RPMI-1640 media supplemented with 2 mM L-glutamine, 100 U/ml penicillin and 100 μg/ml streptomycin (GPS; Sigma-Aldrich, Dorset, UK), phenol red free or phenol red containing RPMI-1640 media supplemented with GPS and 10% heat-inactivated fetal calf serum [HI-FCS; hereafter referred to as complete medium (CM); Sigma-Aldrich], glucose free RPMI-1640 media supplemented with GPS (Gibco, Fisher Scientific UK Ltd, Loughborough, UK), Hank's balanced salt solution (HBSS) supplemented with calcium and magnesium (hereafter referred to as HBSS^+/+^; Gibco, Life Technologies, Cheshire, UK) or HEPES buffer containing 1 mM Ca^2+^.

For mtDAMPs and mtDNA experiments, neutrophils were pre-treated for 15 min (37°C/5% CO_2_) with 40 or 100 μg/ml mtDAMPs or mtDNA prior to secondary stimulation. Prior to inclusion in transmigration, lactate and ROS assays, neutrophils were pelleted, supernatants removed, and cells resuspended in specified media. To inhibit FPR-1 signaling, neutrophils were treated for 60 min (37°C/5% CO_2_) with 2.5 μM cyclosporin H (CsH; Abcam, Cambridge, UK) or vehicle control, prior to mtDAMP stimulation. For compound C experiments, neutrophils were treated for 60 min with 200 μM compound C (Sigma-Aldrich) or vehicle control prior to mtDAMP and PMA treatment. To inhibit calcium-calmodulin-dependent protein kinase kinases (CaMKKs), neutrophils were incubated for 60 min (37°C/5% CO_2_) with 2.5 μM STO-609 (Sigma-Aldrich) or vehicle control prior to mtDAMP treatment. To induce AMP-activated protein kinase (AMPK) signaling, neutrophils were treated for 60 min with 1 mM AICAR (Sigma-Aldrich) prior to PMA stimulation.

### *Ex vivo* NET Formation

Neutrophils (2 × 10^5^ in phenol red free or phenol red containing RPMI + GPS or glucose free RPMI-1640 media supplemented with GPS) were stimulated with 25 nM PMA (Sigma-Aldrich) for 3 h at 37 °C/5% CO_2_. Post-stimulation, supernatants were collected and centrifuged at 2,200 x g for 10 min at 4°C, after which the DNA content of cell-free supernatants was analyzed. Briefly, 100 μl aliquots of cell-free supernatant were incubated with 1 μM SYTOX Green dye (Life Technologies) for 10 min at RT. Fluorescence was measured using a BioTek Synergy 2 fluorometric plate reader (NorthStar Scientific Ltd, Sandy, UK) with excitation and emission set at 485 and 528 nm respectively. In our trauma-based studies, DNA quantification was performed using a λ-DNA standard curve (Fisher Scientific) with PMA-induced NET generation presented as DNA concentration after subtracting the readings obtained from untreated controls. For mtDAMP experiments, background fluorescence values acquired from SYTOX Green staining of mtDAMPs in the absence of neutrophils were subtracted from test readings, with NET production expressed as a fold increase above untreated controls.

### Visualization of NETs by Fluorescence Microscopy

2 × 10^5^ neutrophils in phenol red free or phenol red containing RPMI + GPS or glucose free RPMI-1640 media supplemented with GPS were seeded onto glass coverslips and incubated for 30 min at 37°C/5% CO2 to allow for cell adherence. Following a 3 h stimulation with 25 nM PMA (37°C, 5% CO2), samples were fixed for 30 min with 4% paraformaldehyde (37°C, 5% CO2), washed three times in phosphate buffered saline (PBS) and permeabilised with 0.1% Triton X-100 (Sigma-Aldrich). DNA was then stained with 1 μM SYTOX Green dye for 5 min, after which slides were washed once in PBS, mounted in fluoromount medium and visualized using a LEICA DMI 6000 B microscope (LEICA, Milton Keynes, UK) at x20 or x40 objective.

### ROS Production

For *ex vivo* analysis of neutrophils isolated from trauma patients, ROS generation was assessed by lucigenin-amplified chemiluminescence. The effect of mtDAMP pre-treatment on ROS production was examined using luminol-amplified chemiluminescence. In both instances, 100 μl aliquots of neutrophils (1 × 10^6^/ml in HBSS^+/+^) were dispensed into wells of a 96-well white-bottomed flat plate (BD Biosciences), pre-coated with PBS/2% BSA, that contained 25 μl of luminol (pH 7.3; final concentration 100 μM; Sigma-Aldrich) or lucigenin (final concentration 200 μM; Sigma-Aldrich) and 50 μl HBSS^+/+^. Neutrophils were then stimulated with 25 nM PMA or vehicle control, after which ROS generation was assessed at 1 min intervals for 180 min using a Berthold Centro LB 960 luminometer (Berthold Technologies, Hertfordshire, UK). Experiments were performed in quadruplicate, with ROS production measured as relative light units and calculated as area under the curve (AUC).

### Measurement of Lactate Concentration in Cell-Free Culture Supernatants

Neutrophils (2 × 10^6^ in phenol red free RPMI + GPS) were stimulated for 1, 2, or 3 h (37°C, 5% CO_2_) with 25 nM PMA or vehicle control. At each time-point, cell-free supernatants were harvested (800 x g, 5 min, 4°C) and samples stored at −80°C prior to analysis. Lactate concentration in 25 μl aliquots of supernatant was determined using a commercially available lactate assay kit according to manufacturer's instructions (Sigma-Aldrich).

### Glucose Uptake Assay

Following a 15 min rest period at 37°C/5% CO_2_, neutrophils (1 × 10^6^ in RPMI-1640 media without glucose) were stimulated for 60 min (37°C/5% CO_2_) with 25 nM PMA or vehicle. With 10 min of the stimulation period remaining, the fluorescent glucose analog 2-N-7-nitrobenzen-2oxa-1,3-diazol-4-yl amino-2-deoxyglucose (2-NBDG; Thermo Fisher) at a final concentration of 100 μM was added. Post-incubation, samples were washed and cells re-suspended in glucose free RPMI in preparation for flow cytometric analysis, which was performed on a ${\text{CyAn}}_{\text{ADP}}^{\text{TM}}$ bench top cytometer (Dako, Cambridgeshire, UK). Ten thousand neutrophils were collected and FL1 mean fluorescence intensity values recorded.

### Neutrophil Transmigration

Neutrophils (1 × 10^7^/ml) in HEPES buffer containing 1 mM Ca^2+^ were incubated for 30 min in a 37°C water bath with 3 μg/ml calcein-acetoxmethyl ester (calcein-AM, Fisher Scientific), after which cells were pelleted, supernatants removed and neutrophils re-suspended at 1 × 10^7^/ml in phenol red free CM. A total of 1 × 10^6^ neutrophils were dispensed into the upper chambers of polycarbonate membrane cell culture inserts with 3 μM pores (Corning, New York, USA) that had been pre-loaded into wells of a 24-well flat bottomed plate (BD Biosciences) containing pre-warmed phenol red free CM and 1 nM LTB_4_ (R and D Systems, Abingdon, UK). Following a 90 min incubation at 37°C, cell culture inserts were removed and plates read immediately for calcein fluorescence using a BioTek Synergy 2 fluorometric plate reader with excitation and emission set at 485 and 528 nm respectively. Fluorescence intensities were converted into neutrophil numbers via the use of a standard curve that was generated from calcein-AM loaded neutrophils that had been incubated alongside the test samples in the conditions described above. The number of neutrophils measured in media in which no chemokine was added was subtracted from the numbers calculated for wells that contained 1 ng/ml LTB_4_ in order to determine specific chemokine-mediated migration.

### Assessment of Neutrophil Phenotype

Freshly isolated neutrophils (1 × 10^5^ in CM) were stimulated with 100 μg/ml mtDAMPs or vehicle control for 15 min at 37°C in a humidified 5% CO_2_ atmosphere. Post–treatment, samples were stained on ice for 20 min with the following mouse anti-human monoclonal antibodies or their concentration-matched isotype controls: 2 μg/ml fluorescein isothiocyanate (FITC)-labeled CD62L (clone DREG56; eBioscience, Hatfield, UK); 1 μg/ml CXCR1-FITC (clone eBIO8F1-1-4; eBioscience); 0.5 μg/ml R-phycoerythrin (PE)-labeled CXCR2-PE (clone eBio5E8-C7-F10; eBioscience) or 2.5 μg/ml allophycocyanin (APC)-labeled CD11b (clone ICRF44, BioLegend, London, UK). Post incubation, cells were pelleted (250 x g, 5 min, 4°C), supernatants discarded and neutrophils washed once in PBS/1%BSA. Following resuspension in PBS, samples were transferred to polypropylene FACS tubes for flow cytometric analysis, which was performed on an AccuriC6^TM^ bench top cytometer (BD Biosciences). Ten thousand neutrophils, gated according to their forward scatter (FS)/sideward scatter (SS) properties, were acquired for analysis, where receptor expression was measured as median fluorescence intensity (MedFI).

### Cell Signaling Measurements

To determine signaling through AMPK and MAPK pathways, cell lysates prepared from 2 × 10^6^ resting neutrophils, 1 × 10^6^ neutrophils stimulated with either 25 nM PMA or 100 μg/ml mtDAMPs for 2–90 min (37°C/5% CO_2_), or 2 × 10^6^ neutrophils stimulated with 100 μg/ml mtDAMPs for 5 min following 1 h pre-treatment with 2.5 μM STO-609 or 2.5 μM CsH were separated on 10 or 12% SDS-polyacrylamide gels. Following protein transfer to polyvinylidene difluoride membranes (Bio-Rad, Hertfordshire, UK), blots were probed overnight at 4°C with rabbit anti-human antibodies (Cell Signaling Technology, Massachusetts, USA) directed against phosphorylated AMPK (pAMPK), phosphorylated ERK1/2 (pERK1/2), phosphorylated P38 (pP38), lactate dehydrogenase A (LDHA), or pyruvate kinase (PKM2). Post incubation, membranes were washed in tris-buffered saline containing 0.001% tween (TBST) and incubated for 1 h at RT with a goat anti-rabbit secondary antibody conjugated to horse radish peroxidase (HRP; diluted 1:4000 in TBST; GE Healthcare, Buckinghamshire, UK). HRP activity was detected using enhanced chemiluminescence (Bio-Rad). To confirm equal loading of proteins, blots were probed with antibodies against total ERK 1/2, total P38 (1:1000; Cell Signaling Technology), or β-actin (1:5000, GeneTex, California, USA). Densitometry analysis was performed using Image J software (National Institutes of Health, Bethesda, MD, USA).

### Enzyme-Linked Immunosorbent Assays (ELISAs)

Serum was prepared from blood collected into BD vacutainers containing z-serum clotting activator. Following a 30 min incubation at RT, blood samples were centrifuged at 1,620 x g for 10 min at 4°C, after which serum was aliquoted and stored at −80°C until analyzed. ELISAs to measure serum concentrations of HMGB-1 (IBL International, Hamburg, Germany), mitochondrial encoded NADH dehydrogenase 6 (ND6; MyBioSource, San Diego, California, USA) and IL-33 (R and D Systems) were performed in accordance with manufacturer's instructions.

### Statistical Analyses

Statistical analyses were performed using GraphPad Prism® software (GraphPad Software Ltd, California, USA). Data distribution was examined using the Kolmogorov-Smirnov or Shapiro-Wilk normality test. For data that followed a normal distribution, paired student *t*-tests, a repeated measures ANOVA with Bonferroni multiple comparison *post hoc* test or a one way ANOVA with Dunnett's multiple comparison *post hoc* test were performed. For non-normally distributed data, a Wilcoxon matched-pairs signed rank test, a Friedman test with Dunn's multiple comparison *post hoc* test or a Kruskal-Wallis with Dunn's multiple comparison *post hoc* test was performed. For box and whisker plots, whiskers represent minimum and maximum values. Statistical significance was accepted at *p* ≤ 0.05.

## Results

### Patient Enrolment and Demographics

1,070 adult trauma patients were screened for study inclusion, with 87 subjects enrolled into the study (Supplementary Figure 1). Of these, 62 patients with a mean age of 44 years (range 19–95 years) and mean injury severity score of 26 (range 9–57) had their immune function analyzed (Table 1). The mean time of pre-hospital blood sampling was 39 min post-injury (range 13–59 min).

**Table 1:** Cohort demographics.

	**Patients (*n* = 62)**
Age, years	44 (19–95)
Male, *n* (%)	56 (90)
Time to pre-hospital sample, minutes post-injury	39 (13–59)
ISS	26 (9–57)
NISS	38 (9–75)
**AIS**	
Head, *n* (%)	24 (48)
Face, *n* (%)	17 (34)
Chest, *n* (%)	29 (58)
Abdomen, *n* (%)	14 (28)
Spine, *n* (%)	19 (38)
Pelvis, *n* (%)	8 (16)
Limbs, *n* (%)	28 (56)
Other, *n* (%)	6 (12)
**MECHANISM OF INJURY**	
Fall, n (%)	10 (16)
A/P, *n* (%)	13 (21)
Blunt, *n* (%)	2 (3)
RTC, *n* (%)	37 (60)
**OUTCOMES**	
ICU-free days	22 (0–30)
Hospital-free days	13 (0–29)
Mortality, *n* (%)	8 (13)

*Data are expressed as mean (range) unless indicated otherwise*.

*The number of data points for each clinical variable are: ISS and NISS, n = 48; AIS scores, n = 50; ICU and hospital free days, n = 53*.

*ICU-free days and hospital-free days were calculated by 30 minus the number of days the patient stayed in hospital)*.

*A/P, Assault/penetrating; AIS, Abbreviated injury scale; ISS, Injury severity score; ICU, Intensive care unit; NISS, New injury severity score; RTC, Road traffic collision*.

### PMA-Induced NET Production Is Impaired Post-trauma

**Figure 1:**
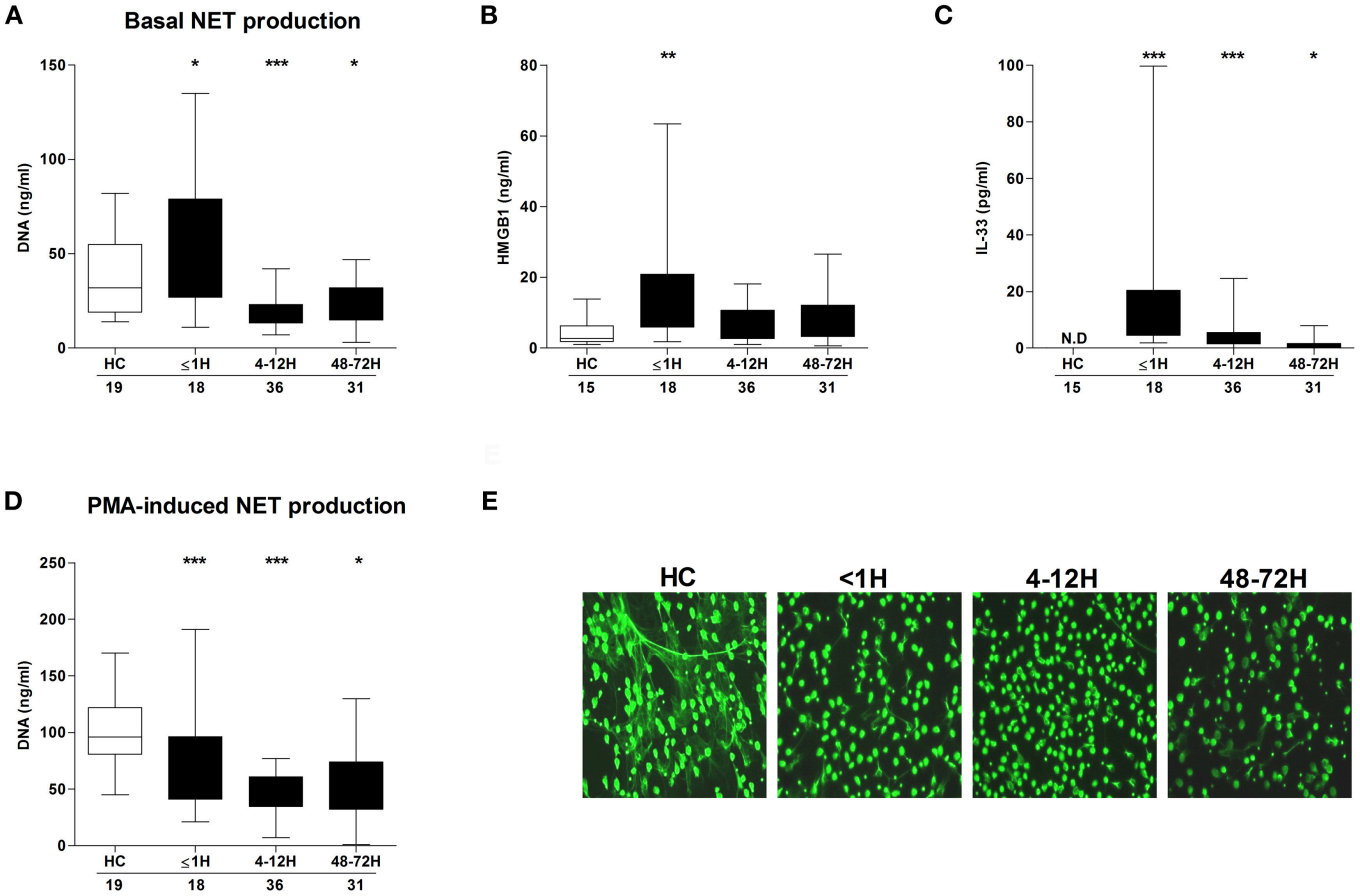
Effect of traumatic injury on PMA-induced NET formation. **(A)** Basal NET generation by resting neutrophils isolated from healthy controls (HC) and trauma patients as assessed by DNA concentration in cell free supernatants following a 3 h *in vitro* culture. **(B)** HMGB-1 and **(C)** IL-33 concentrations in serum samples from HC and trauma patients. IL-33 levels were undetectable (N.D) in serum samples from HC. **(D,E)** Following a 3 h *in vitro* stimulation with PMA, NET production by neutrophils from HC and trauma patients was compared by measuring DNA concentration in cell free supernatants **(D)** and fluorescence microscopy **(E)**. For supernatant analysis, number of samples are shown below each time-point. For microscope images, HC (*n* = 12), ≤1 h (*n* = 6), 4–12 h (*n* = 6), and 48–72 h (*n* = 8). ^*^p < 0.01, ^**^p < 0.001, ^***^p < 0.0001 vs. HC.

Compared to neutrophils isolated from HCs, neutrophils acquired from trauma patients within 1 h of injury exhibited significantly enhanced basal NET generation (Figure 1A), a hyperactivity that was accompanied by significantly elevated serum concentrations of HMGB-1 (Figure 1B) and IL-33 (Figure 1C). By the 4–12 and 48–72 h post-injury time points, a significant reduction in basal NET production was observed (Figure 1A). In response to stimulation with PMA, patient neutrophils released significantly less DNA at all three sampling time-points when compared to HCs (Figure 1D). Fluorescence microscopy confirmed the impairment in NET generation (Figure 1E).

### ROS Production in Response to PMA Stimulation Is Reduced in the Acute Post-injury Phase

**Figure 2:**
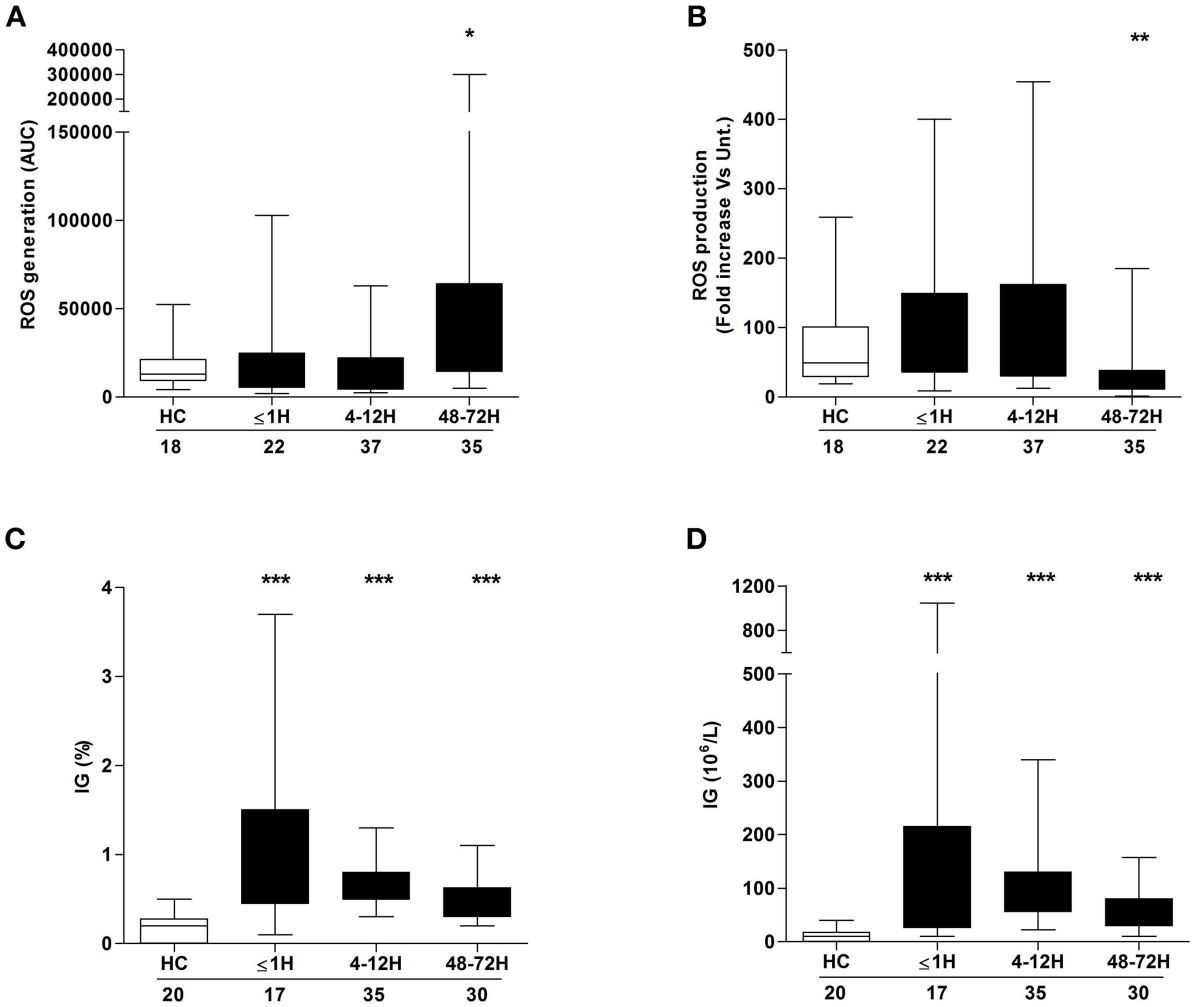
Neutrophil ROS production and immature granulocyte count post-injury. Comparison of basal **(A)** and PMA-induced **(B)** ROS production by neutrophils isolated from healthy controls (HC) and trauma patients. Data are presented as area under the curve (AUC) **(A)** or fold increase above vehicle treated controls **(B)**. **(C,D)** Frequency **(C)** and absolute number **(D)** of immature granulocytes (IG) in peripheral blood samples from healthy controls (HC) and trauma patients. Number of samples analyzed are shown below each time-point. ^*^p < 0.01, ^**^p < 0.001, ^***^p < 0.0001 vs. HC.

ROS generation is a non-redundant event in NET formation (17). Having observed trauma-induced alterations in both basal and stimulated NET formation, we examined the effect of injury on ROS production. In the absence of stimulation, patient neutrophils isolated 48–72 h post-injury exhibited significantly enhanced ROS production when compared to the response of neutrophils from HCs (Figure 2A). No difference in basal ROS generation was seen between HCs and patient neutrophils acquired ≤ 1 h or 4–12 h post-injury (Figure 2A). In response to PMA stimulation, there was a significant reduction in ROS production, relative to HCs, for neutrophils isolated from patients only at the 48–72 h post-injury time point (Figure 2B).

### Traumatic Injury Results in an Immediate and Sustained Elevation in the Frequency and Absolute Number of Circulating IGs

Compared to their mature counterparts, immature neutrophils exhibit impaired *ex vivo* NET production and reduced ROS production upon stimulation with inflammatory agonists (1, 27). Relative to the values recorded for HCs, trauma patients presented, at all sampling time points, with a significantly elevated frequency (Figure 2C) and absolute number (Figure 2D) of circulating IGs.

### Traumatic Injury Is Associated With Impaired MAPK Signaling

**Figure 3:**
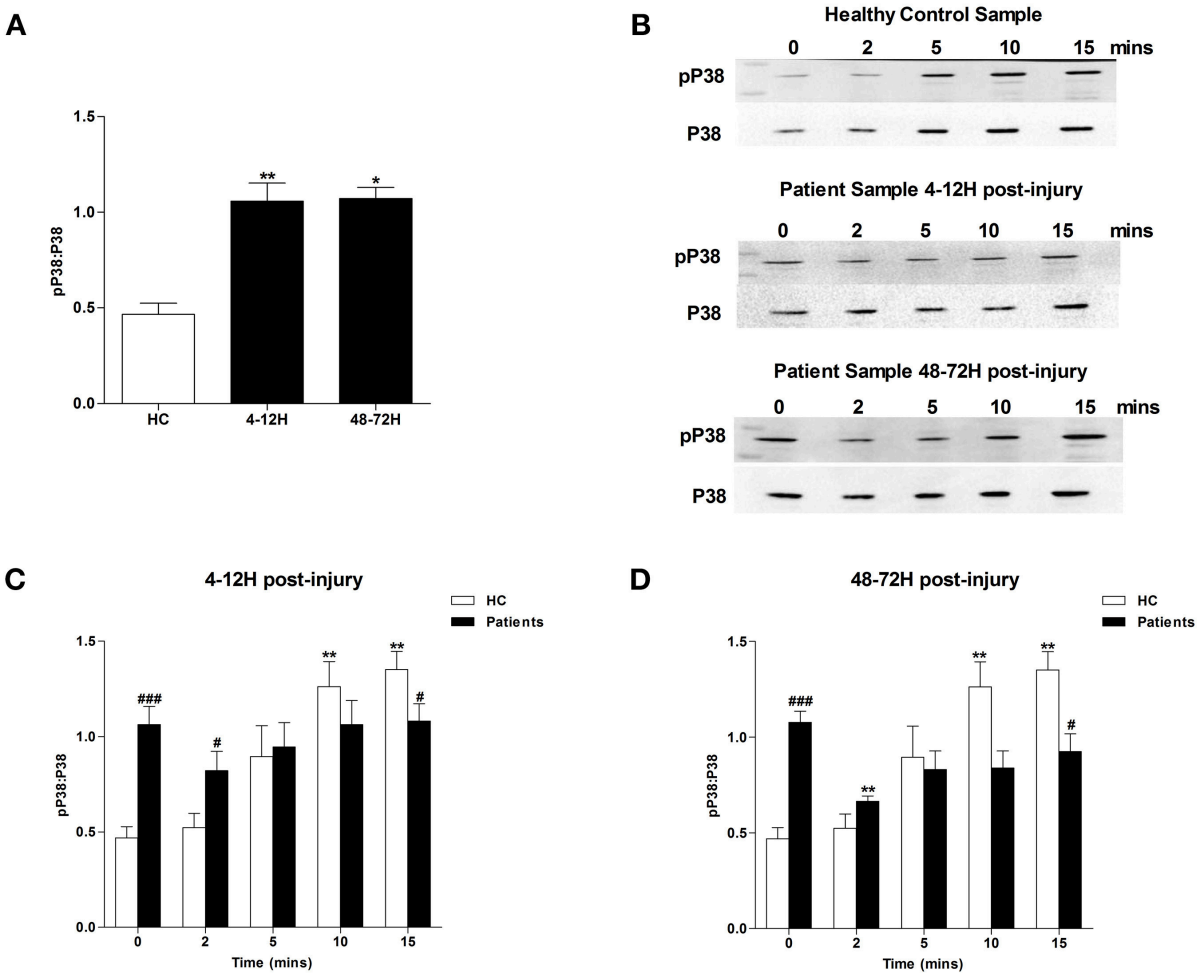
Traumatic injury results in impaired PMA-induced activation of p38 MAPK. Phosphorylation status of P38 in resting and PMA-stimulated neutrophils isolated from healthy controls (HC) and trauma patients 4–12 and 48–72 h post-injury. Data are presented as representative Western blots **(B)** and densitometry analysis of p38 phosphorylation in resting **(A)** or PMA-stimulated neutrophils at the 4–12 h **(C)** and 48–72 h **(D)** post-injury time points. HC (*n* = 7), 4–12 h (*n* = 9), and 48–72 h (*n* = 4). For **(A)** ^*^p < 0.01, ^**^p < 0.001 vs. HC. For **(C)** and **(D)** ^**^p < 0.001 vs. Time 0, ^#^*p* < 0.01, ^###^*p* < 0.0001 vs. HC sample at matched time point.

**Figure 4:**
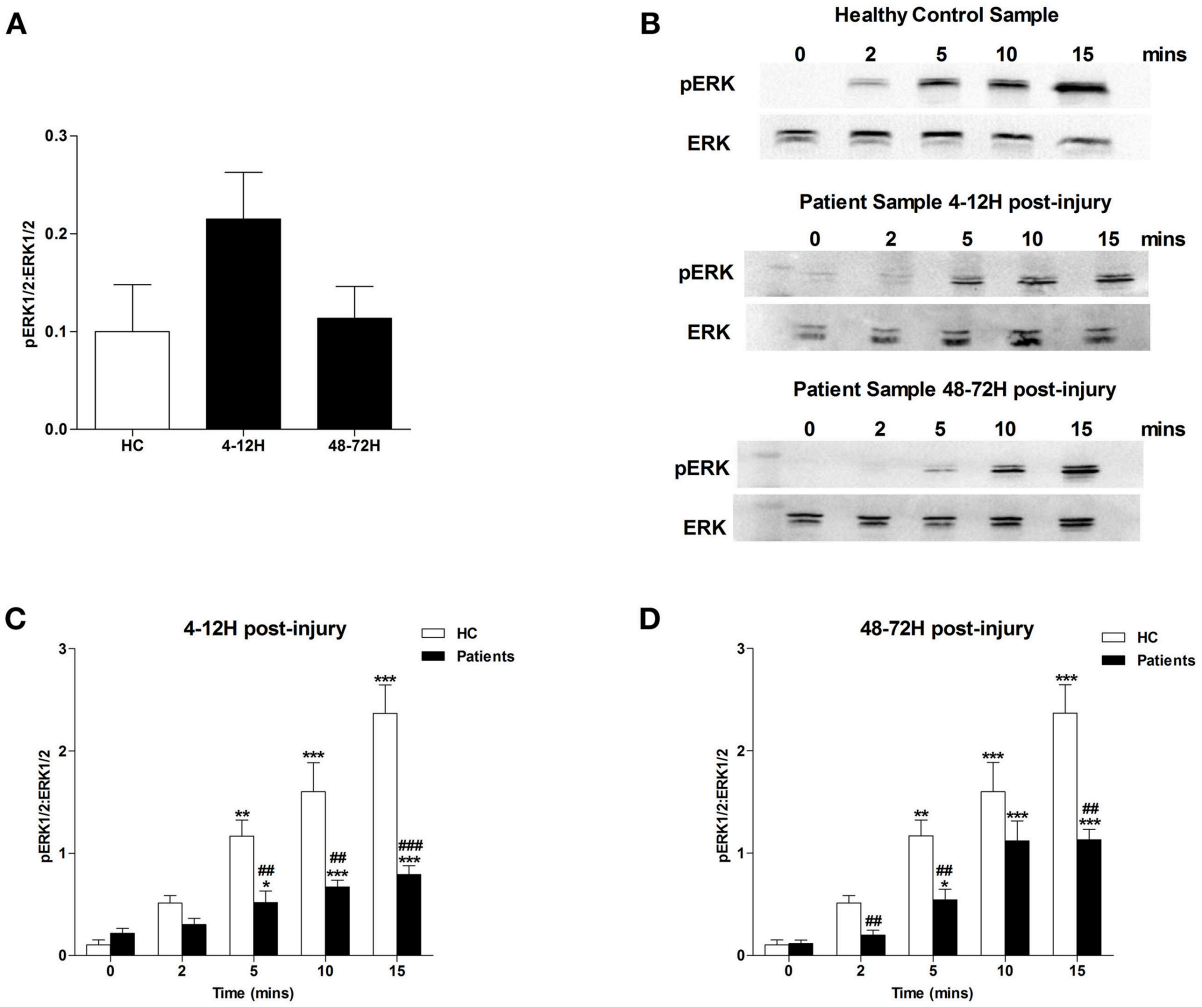
Traumatic injury results in impaired PMA-induced activation of ERK MAPK. Phosphorylation status of ERK 1/2 in resting and PMA-stimulated neutrophils isolated from healthy controls (HC) and trauma patients 4–12 and 48–72 h post-injury. Data are presented as representative Western blots **(B)** and densitometry analysis of ERK phosphorylation in resting **(A)** or PMA-stimulated neutrophils at the 4–12 h **(C)** and 48–72 h **(D)** post-injury time points. HC (*n* = 4), 4–12 h (*n* = 8), and 48–72 h (*n* = 6). For **(C)** and **(D)** ^*^p < 0.01, ^**^p < 0.001, ^***^p < 0.0001 vs. Time 0, ^##^*p* < 0.001, ^###^*p* < 0.0001 vs. HC sample at matched time point.

MAPK signaling is a prerequisite for PMA-induced NET production (20). Due to the significant lymphocytosis that occurs within minutes of traumatic injury (3), and the small blood volume collected from patients at the scene of injury, we were unable to isolate a sufficient number of neutrophils from pre-hospital blood samples to examine MAPK signaling. However, we found neutrophils isolated from patients 4–12 and 48–72 h post-injury exhibited significantly increased phosphorylation of P38 MAPK (Figure 3A) but not ERK1/2 (Figure 4A) in the absence of exogenous stimulation.

In response to treatment with PMA, neutrophils obtained from HCs exhibited a significant increase in P38 phosphorylation (Figures 3B–D). In contrast, no significant PMA-induced increase in P38 phosphorylation was observed for neutrophils isolated from trauma patients 4–12 or 48–72 hours post-injury (Figures 3B–D). Compared to untreated cells, neutrophils isolated from HCs and trauma patients at the 4–12 and 48–72 h post-injury time points displayed a significant increase in ERK1/2 phosphorylation following 5, 10, and 15 min of PMA stimulation (Figures 4B–D). However, across these three stimulation time points, the degree of ERK1/2 phosphorylation was significantly greater in neutrophils isolated from HCs (Figures 4B–D).

### Altered Neutrophil Glucose Uptake and Metabolism Post-trauma

**Figure 5:**
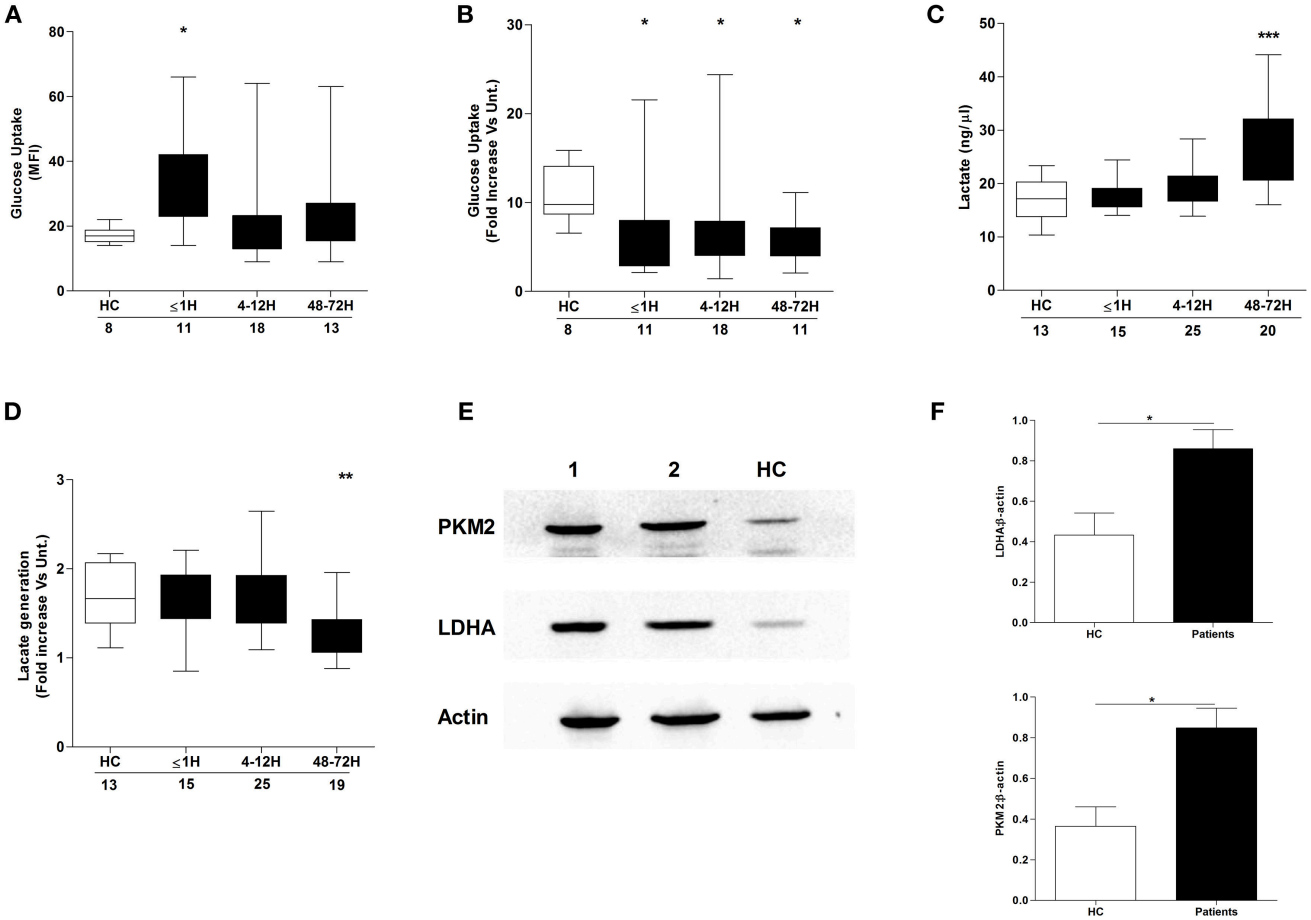
Effect of traumatic injury on neutrophil glucose uptake and metabolism. Glucose uptake by basal **(A)** or PMA-stimulated **(B)** neutrophils isolated from healthy controls (HC) and trauma patients. MFI, Mean fluorescence intensity. **(C,D)** Comparison of lactate concentration in cell-free supernatants collected from resting **(C)** or PMA stimulated **(D)** neutrophils isolated from HC and trauma patients following a 3 h *in vitro* culture. Number of samples analyzed are indicated below each time-point. ^*^p < 0.01, ^**^p < 0.001, ^***^p < 0.0001 vs. HC. **(E,F)** Expression of the glycolytic enzymes pyruvate kinase (PKM2) and lactate dehydrogenase A (LDHA) in resting neutrophils isolated from HC (*n* = 5) and trauma patients 48–72 h post-injury (*n* = 14). Data are presented as representative Western blots **(E)** and densitometry analysis of collated data for LDHA (**F**, top panel) and PKM2 (**F**, bottom panel). ^*^p < 0.01 vs. HC.

Confirming the results of a recent study that demonstrated a necessity for exogenous glucose in PMA-induced NET production (21), we found neutrophils cultured in glucose free media released significantly less DNA upon PMA stimulation than neutrophils stimulated in glucose containing media (Supplementary Figure 2). Based on our observation of a trauma-induced impairment in *ex vivo* NET generation following PMA treatment, we investigated the effect of injury on neutrophil glucose uptake. Using the fluorescent glucose analog 2-NBDG, enhanced basal glucose uptake was recorded for neutrophils isolated from trauma patients within 1 h of injury (Figure 5A), but in response to PMA stimulation, a significant trauma-induced impairment in neutrophil glucose uptake was seen at all sampling time points (Figure 5B).

We next examined whether injury impacted upon glucose metabolism, a non-redundant step in NET formation triggered by PMA stimulation (21). Using lactate production as a marker of neutrophil glycolytic activity, we measured lactate concentrations in supernatants collected from resting and PMA-stimulated neutrophils following a 3 h *in vitro* culture. Compared to HCs, neutrophils isolated from trauma patients at the 48–72 h post-injury time-point exhibited enhanced basal (Figure 5C) but impaired PMA-induced lactate production (Figure 5D). The increase in basal lactate generation was accompanied by a significant up-regulation in the expression of the glycolytic enzymes pyruvate kinase and lactate dehydrogenase A (Figures 5E,F).

### Neutrophils Pre-treated With mtDAMPs Exhibit Impaired NET Production but Enhanced ROS Generation Upon Secondary Stimulation

**Figure 6:**
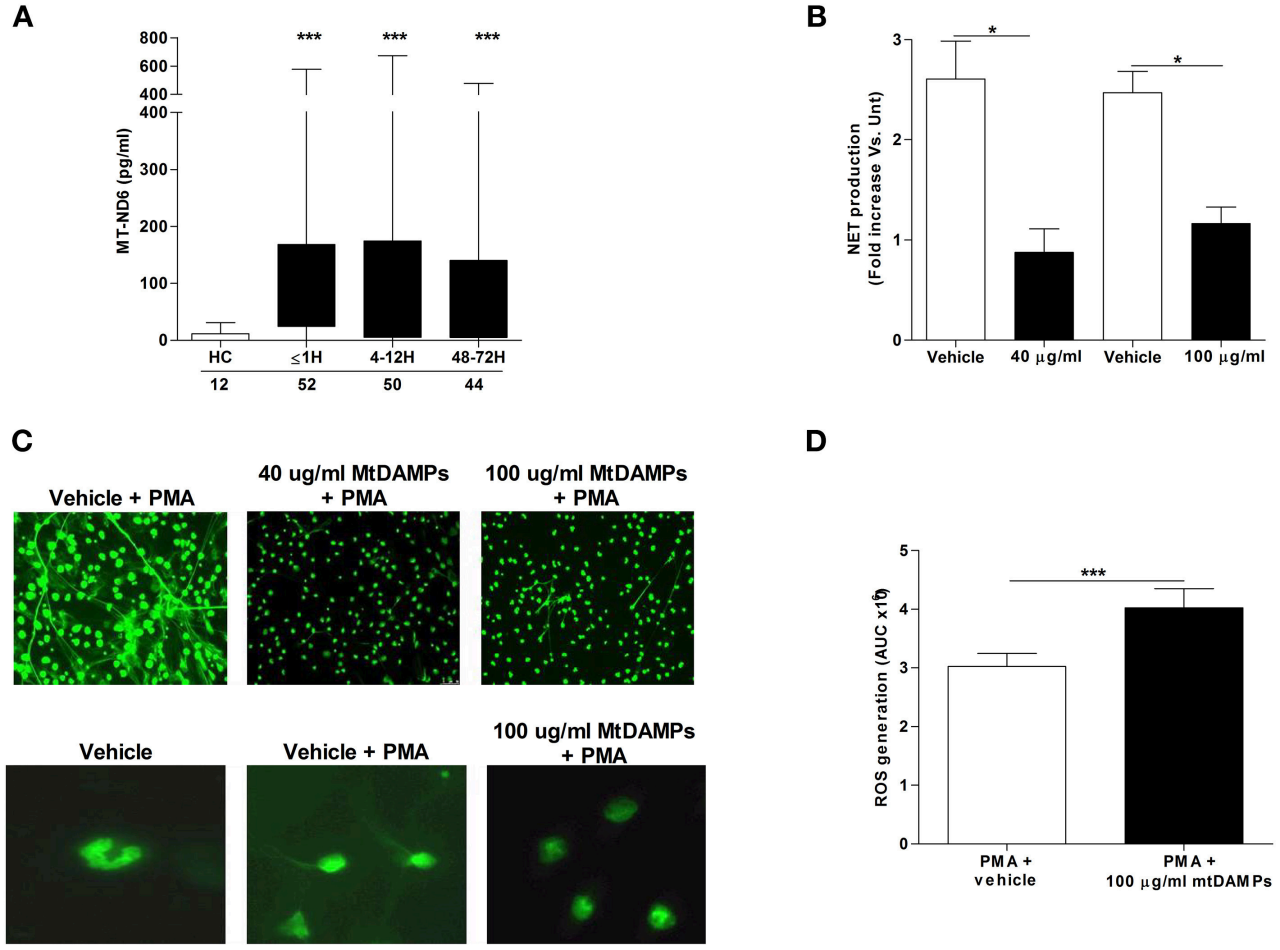
Effect of mtDAMP pre-treatment on neutrophil NET generation. **(A)** Serum concentrations of the mitochondrial-derived N-formylated peptide ND6 in peripheral blood samples acquired from healthy controls (HC) and trauma patients. Number of samples are indicated below each time-point. ^***^p < 0.0001 vs. HC. **(B)** NET production by PMA stimulated neutrophils pre-treated with 40 or 100 μg/ml mtDAMPs was assessed by measuring DNA content of cell-free supernatants (*n* = 5). ^*^p < 0.01 vs. Vehicle. **(C)** MtDAMP-induced inhibition of NET generation was confirmed by fluorescence microscopy (*n* = 5; top panel x20 magnification; bottom panel x40 magnification). **(D)** ROS generation by neutrophils pre-treated with 100 μg/ml mtDAMPs or vehicle control was measured in response to stimulation with 25 nM PMA using luminol-based chemiluminescence (*n* = 10). Data are presented as area under the curve (AUC) and are mean ± SEM. ^***^p < 0.0001 vs. PMA.

Compared to the levels measured in samples from HCs, serum concentrations of the mitochondrial-derived protein ND6 were significantly increased in patients at all post-injury time points, confirming the release of mtDAMPs after trauma (Figure 6A). Demonstrating the immune stimulatory properties of mtDAMPs, we measured significantly reduced CD62L, CXCR1, and CXCR2 expression as well as increased CD11b density on the surface of mtDAMP treated neutrophils (Supplementary Table 1). These changes in neutrophil surface phenotype were accompanied by activation of ERK 1/2 MAPK signaling (Supplementary Figure 3A). The emerging concept of mtDAMP-induced tolerance of neutrophil function is based in part on experimental data that has shown prior activation of neutrophils with bacterial-derived N-formylated peptides results in impaired migration upon secondary stimulation (10). Confirming these findings, we found that neutrophils pre-treated with 40 or 100 μg/ml preparations of whole mtDAMPs exhibited significantly reduced transmigration toward the chemokine LTB_4_ (Supplementary Figure 3B). In contrast, no impairment in migration was witnessed for neutrophils pre-treated with 100 μg/ml of purified mtDNA (Supplementary Figure 3C).

To determine whether prior mtDAMP treatment influenced PMA-induced NET production, fluorometric analysis was performed on cell-free supernatants collected from cultures of PMA stimulated neutrophils that had been pre-treated with mtDAMPs or vehicle control. Analysis revealed neutrophils pre-exposed to 40 or 100 μg/ml mtDAMPs released significantly less DNA following a 3 h stimulation with PMA than vehicle-treated controls (Figure 6B). Fluorescence microscopy confirmed this mtDAMP-induced inhibition of PMA-induced NET generation (Figure 6C). Interestingly, focusing upon neutrophils pre-treated with 100 μg/ml mtDAMPs, images revealed that despite a significant reduction in NET formation, the cells had lost their multi-lobed nuclear morphology following stimulation with PMA, presenting with decondensed nuclear material (Figure 6C). In contrast to whole mtDAMP preparations, neutrophils pre-treated with 40 μg/ml of purified mtDNA prior to PMA stimulation showed no impairment in NET production (data not shown). Interestingly, ROS production, which is a prerequisite for NET formation, was significantly higher upon secondary PMA stimulation for neutrophils pre-treated with 100 μg/ml mtDAMPs (Figure 6D).

### AMPK Is Activated by mtDAMP Treatment and Inhibition of AMPK Partially Ameliorates the mtDAMP-Induced Reduction in NET Formation

**Figure 7:**
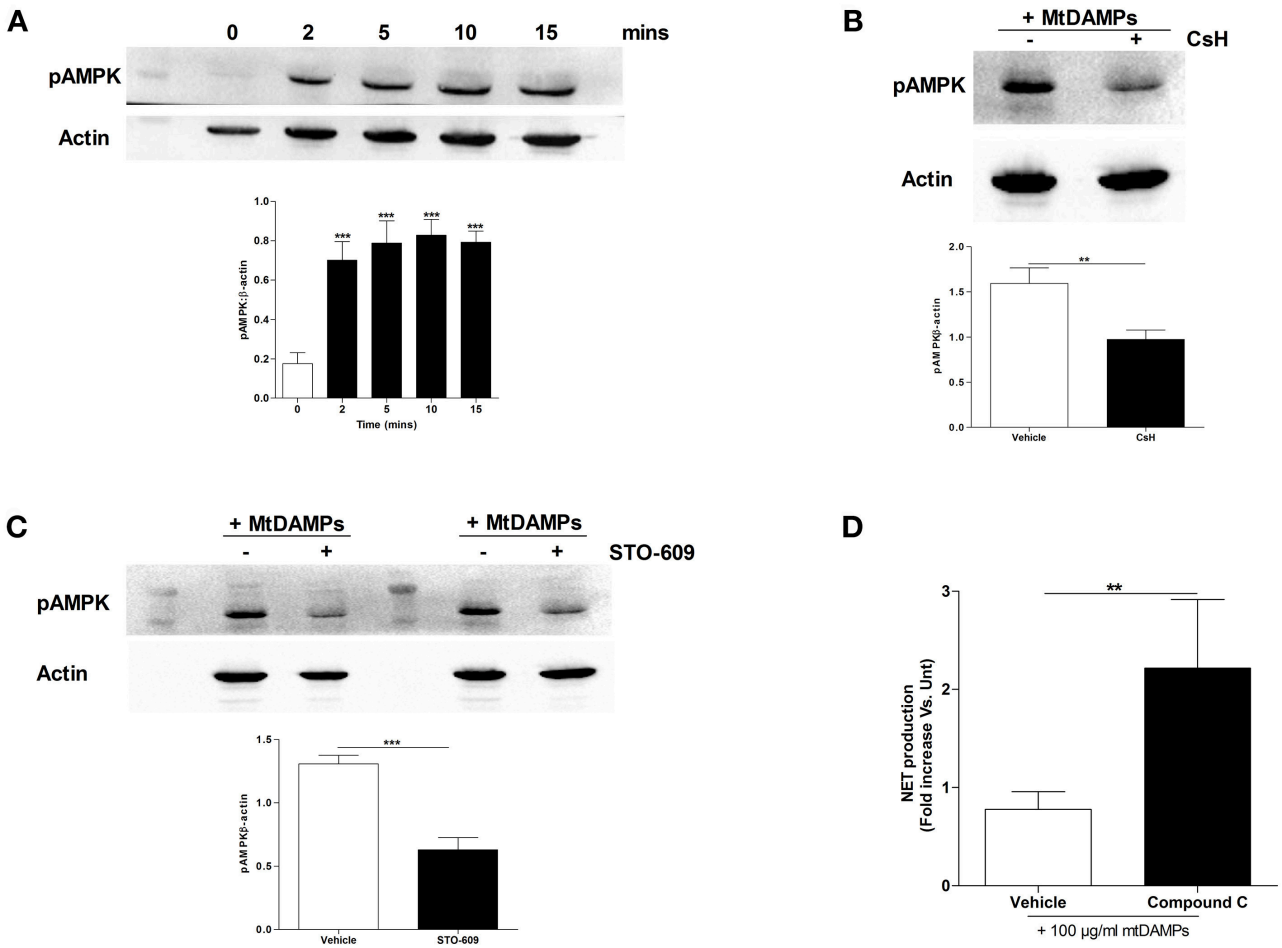
Treatment of neutrophils with mtDAMPs results in phosphorylation of AMPK. **(A)** Whole cell lysates prepared from purified neutrophils stimulated for 2–15 min with 100 μg/ml mtDAMPs were screened for phosphorylated AMPK. Western blot in top panel is representative of 4 independent experiments. For densitometry analysis ^***^p < 0.0001 vs. 0 min. **(B)** AMPK phosphorylation in neutrophils treated for 1 h with the FPR-1 antagonist Cyclosporin H (CsH) or **(C)** or the CaMKK inhibitor STO-609 prior to a 5 min stimulation with 100 μg/ml mtDAMPs. Blots are representative of 5 **(B)** and 10 **(C)** independent experiments, with densitometric data depicted in the accompanying histogram. ^**^p < 0.001, ^***^p < 0.0001 vs. vehicle. **(D)** Comparison of PMA-induced NET formation by mtDAMP stimulated neutrophils pre-treated with the AMPK inhibitor compound C or vehicle control (*n* = 10). ^**^p < 0.01 vs. PMA treated.

In stimulated T cells, elevated intracellular calcium levels activate AMPK, a recently described negative regulator of PMA-induced NET formation (28, 29). As raised intracellular calcium levels are a feature of mtDAMP treated neutrophils (23), we determined the activation status of AMPK in neutrophils following mtDAMP stimulation. To do this, cell lysates, prepared from neutrophils stimulated for 2, 5, 10, and 15 min with 100 μg/ml mtDAMPs, were probed with a phospho-specific antibody directed against Thr172, a residue within the activation loop of AMPK. As shown in Figure 7A, mtDAMP treatment resulted in an immediate and persistent phosphorylation of residue Thr172. Treating neutrophils with the FPR-1 antagonist CsH prior to mtDAMP stimulation resulted in a significant reduction in AMPK phosphorylation, suggesting that N-formyl peptides drive mtDAMP-induced activation of AMPK (Figure 7B). In antigen challenged T cells, phosphorylation of AMPK requires the activation of calcium-calmodulin-dependent protein kinase kinases (CaMKKs), a class of serine/threonine protein kinases activated by increases in intracellular calcium (28). To investigate whether CaMKKs were involved in mtDAMP-induced phosphorylation of AMPK in neutrophils, we treated neutrophils with the CaMKK selective inhibitor STO-609 prior to mtDAMP stimulation. Compared to vehicle control, a significant impairment in mtDAMP-induced activation of AMPK was detected in neutrophils pre-treated with STO-609 (Figure 7C).

AMPK has recently been shown to be a negative regulator of PMA-induced NET formation (29, 30). To investigate whether AMPK signaling was involved in mtDAMP-mediated suppression of NET formation, we treated neutrophils with compound C, an inhibitor of AMPK, prior to mtDAMP exposure. Compared to vehicle control, significantly greater NET production in response to PMA stimulation was recorded for neutrophils pre-treated with compound C (Figure 7D).

### MtDAMP Pre-treatment Results in Impaired Lactate Generation by Neutrophils Upon Secondary Stimulation With PMA

**Figure 8:**
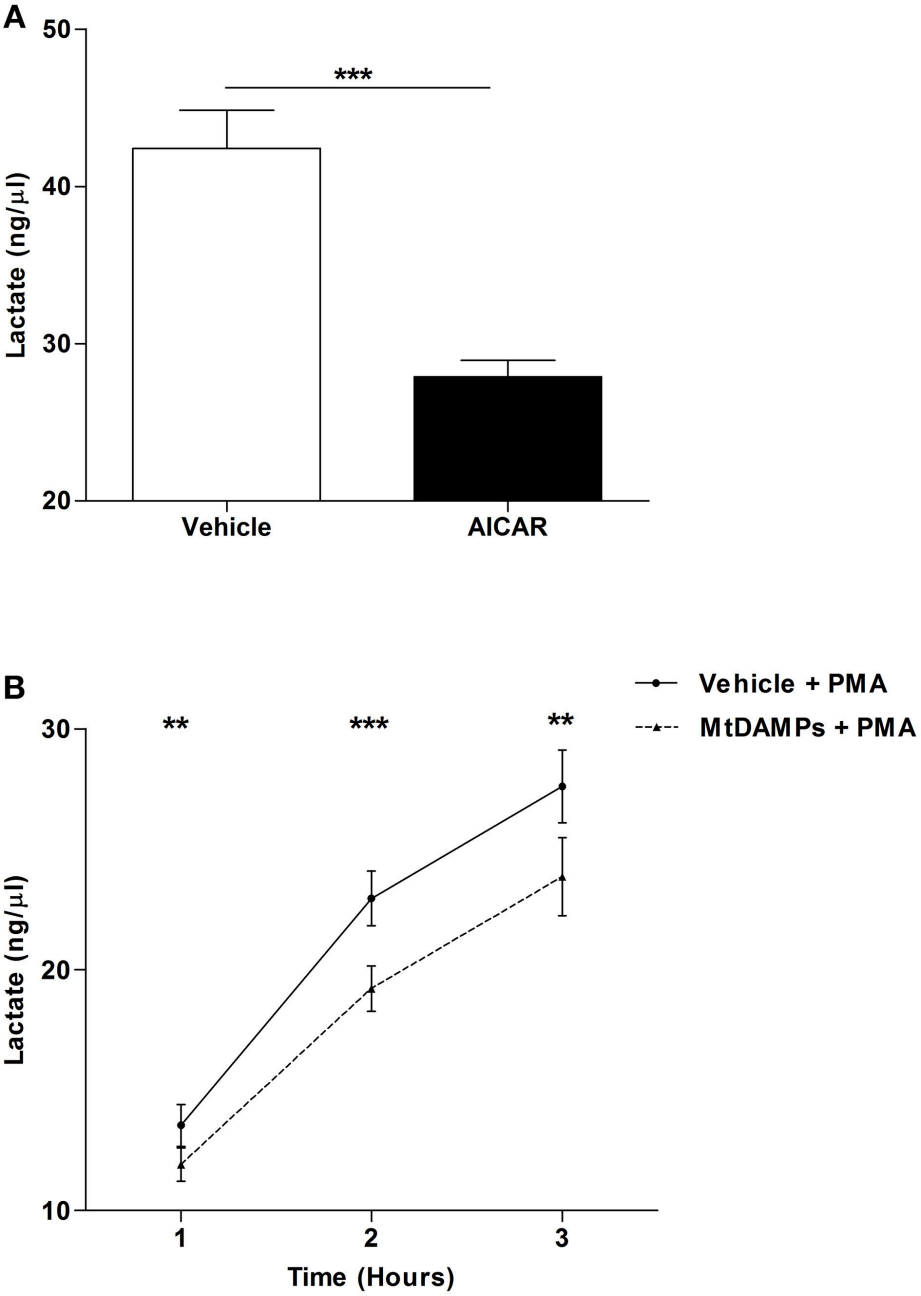
MtDAMP pre-treatment results in impaired neutrophil glycolysis. **(A)** Neutrophils pre-treated for 1 h with 1 mM AICAR or vehicle control were stimulated for 3 h with 25 nM PMA, after which lactate concentrations were measured in cell-free supernatants. Data are mean ± SEM of 12 independent experiments. ^***^p < 0.0001. **(B)** Comparison of lactate concentrations in supernatants collected from neutrophils pre-treated with 100 μg/ml mtDAMPs or vehicle-control and subsequently stimulated with 25 nM PMA for 1, 2, and 3 h. Data are mean ± SEM of 10 independent experiments. ^**^p < 0.001, ^***^p < 0.0001 vs. vehicle.

Confirming results of previous studies that had shown AMPK to be a negative regulator of aerobic glycolysis (31), we measured significantly lower concentrations of lactate in supernatants collected from PMA stimulated neutrophils that had been pre-treated with the AMP mimetic AICAR when compared to vehicle control (Figure 8A). Given that aerobic glycolysis is a key metabolic event in PMA-induced NET formation (21) and our observation of reduced NET generation following PMA stimulation for neutrophils pre-treated with mtDAMPs (Figures 6B,C), we investigated whether AMPK activation triggered by mtDAMP exposure was associated with an impairment in neutrophil glycolysis. Following 1, 2, or 3 h stimulation with PMA, significantly lower lactate concentrations were measured in supernatants collected from neutrophils pre-treated with 100 μg/ml mtDAMPs (Figure 8B).

## Discussion

Here, via the analysis of blood samples acquired from trauma patients within 1 h of injury, we have shown for the first time that major trauma results in an immediate impairment in neutrophil anti-microbial defenses, specifically the formation of NETs. This defect persisted into the acute post-injury phase (4–72 h) and was accompanied by impaired ROS production, MAPK activation and a reduction in both glucose uptake and breakdown. Furthermore, we showed that the trauma-induced reduction in NET formation could be replicated *in vitro* by treating neutrophils isolated from healthy donors with mtDAMPs prior to secondary stimulation. Thus, our data provides support for the emerging concept of mtDAMP-induced tolerance, where the post-injury release of mtDAMPs into the circulation has been proposed to contribute to the neutrophil dysfunction that develops in the aftermath of traumatic injury (10, 22).

In the absence of secondary stimulation, neutrophils obtained from patients within minutes, but not hours, of injury released significantly more DNA into culture supernatants than neutrophils isolated from healthy controls. This immediate trauma-induced increase in basal NET generation is in agreement with the findings of our previous trauma-based study, where in pre-hospital plasma samples, we reported the presence of citrullinated histone H3, a protein that decorates the nuclear DNA backbone of NETs (3, 32). Thus, taken together, our results suggest that neutrophils are immediately exposed to NET-inducing stimuli post-injury. Supporting this proposal, data presented here and in our previous manuscript have shown serum concentrations of known NET inducers, which include TNF-α, IL-8, IL-33, and HMGB-1 are all significantly elevated within minutes of injury (3, 15, 32–34). Of these agonists, the immediate release of HMGB-1 may be particularly pertinent given that within 60 min of *in vitro* co-culture, neutrophils stimulated with this nuclear-derived DAMP have been shown to generate NETs (35). Moreover, as HMGB-1 induced NET formation is independent of ROS generation by NADPH oxidase (15), immediate exposure to this DAMP could explain our observation of enhanced *ex vivo* NET formation by neutrophils isolated from pre-hospital blood samples that exhibited no difference in basal ROS production when compared to neutrophils from HCs. In terms of stimulus-induced NET formation, this is the first study to show quantitatively that trauma results in reduced NET generation to PMA stimulation. We confirmed this impairment, which was evident at all three sampling time points, by fluorescent microscopy, with our images akin to those presented in a previous study that reported a qualitative post-injury reduction in PMA-induced NET production in a much smaller cohort of trauma patients (10).

The processes that mediate PMA-induced NET generation are well-defined, with prominent roles assigned to ROS generation, MAPK activation, glucose uptake and glycolysis (17, 19–21). Across our sampling time points, we observed defects in each of these processes, suggesting that multiple impairments rather than a single aberration underlie the post-injury reduction in NET formation. Whilst our data demonstrating a trauma-induced impairment in neutrophil ROS production has been described previously (1, 3), we are the first to report a post-trauma reduction in MAPK activation, glucose uptake, and metabolism by neutrophils in response to *ex vivo* stimulation. Aside from NET production, other anti-microbial mechanisms of neutrophils utilize glucose. For instance, chemotaxis requires the uptake of exogenous glucose (36), whilst breakdown of endogenous glucose is important for stimulus-induced ROS production and phagocytosis (21, 36). Interestingly, as reported here for NET generation, these three defense strategies have all been shown in *ex vivo* assays to be significantly impaired following trauma (1, 9–11). Thus, the injury-induced reduction we have demonstrated in glucose uptake and metabolism may be a mechanism underlying many facets of post-trauma neutrophil dysfunction. Aside from the changes we found in neutrophil signaling and metabolism, trauma patients presented at all three time points with elevated circulating levels of IGs. Shown *in vitro* to exhibit impaired NET production upon secondary stimulation (27), the immediate and persistent presence of immature cells offers another potential mechanistic explanation for the trauma-induced reduction in NET formation.

Suggesting that traumatic injury modulates cell metabolism, we demonstrated a post-trauma elevation in lactate production by resting neutrophils, an observation that is in agreement with the findings of a previous study. In a cohort of polytrauma patients, Oehler et al. reported a higher glycolytic activity, relative to HCs, for neutrophils isolated from subjects between 48 and 120 h post-injury, an enhancement they attributed to increased expression and activity of pyruvate kinase, a glycolytic enzyme that catalyzes the conversion of phosphoenolpyruvate to pyruvate (37). Here, we confirmed that traumatic injury induces increased expression of pyruvate kinase and showed that this is accompanied by increased expression of lactate dehydrogenase A. Whilst up-regulation of lactate dehydrogenase A, which converts pyruvate to lactate, has been described in transcriptomic analysis of whole blood leukocytes isolated from critically-ill patients (38), this is the first study to demonstrate increased protein expression of lactate dehydrogenase in neutrophils post-injury.

Accompanying the impairment we observed in *ex vivo* NET formation was a significant trauma-induced elevation in the circulating levels of the mitochondrial-derived N-formylated peptide ND6. In a recent study, suppressed chemotactic responses toward CXCL1 and LTB_4_ were reported for neutrophils pre-treated with synthetic ND6 (22), a finding that mirrored results of previous studies where prior exposure to bacterial-derived N-formylated peptides or ND6 respectively was shown to reduce neutrophil migration and ROS production upon secondary stimulation (10, 26). Adding to this growing body of literature that suggests a tolerising effect for mitochondrial-derived peptides on neutrophil function, we demonstrated that neutrophils pre-treated with whole mtDAMP preparations, but not purified mtDNA, exhibited significantly reduced NET production following PMA stimulation.

A striking observation of our NET based assays was that despite an absence of NET production, mtDAMP pre-treated neutrophils lost their distinctive multi-lobed nuclear morphology upon PMA challenge. Interestingly, chromatin decondensation in the absence of DNA release was recently reported for PMA stimulated neutrophils pre-treated with the glycolysis inhibitor 2-deoxy-glucose (21). Using lactate as a readout of glycolytic activity, we measured significantly reduced lactate concentrations in the supernatants of mtDAMP pre-treated neutrophils stimulated with PMA, demonstrating that exposure to mtDAMPs influences the metabolism of immune cells.

We found that exposing neutrophils to mtDAMPs resulted in activation of the serine/threonine protein kinase AMPK. In T cells, increases in intracellular calcium levels promote AMPK phosphorylation through activation of CaMKKs (28). Our data demonstrating a significant reduction in mtDAMP-induced phosphorylation of AMPK in neutrophils pre-treated with the selective CAMMK inhibitor STO-506 indicates this signaling pathway is also activated in stimulated neutrophils. As the only component of mtDAMPs that promotes calcium mobilization in neutrophils (23), signals derived from N-formyl peptides are likely to have driven the mtDAMP-induced phosphorylation of AMPK. Supporting this idea, we observed significantly reduced mtDAMP-induced AMPK phosphorylation in neutrophils pre-treated with CsH, an FPR-1 antagonist that prevents calcium mobilization upon mtDAMP stimulation (23).

Demonstrating that AMPK is a negative regulator of NET formation, significantly enhanced and impaired NET formation has been reported for PMA stimulated neutrophils pre-treated with AMPK inhibitors and activators respectively (29, 30). Supporting these data, we showed significantly greater PMA-induced NET production by neutrophils treated with the AMPK inhibitor compound C prior to mtDAMP exposure. How activation of AMPK inhibits NET formation is currently unknown. Based on published literature, we propose two mechanisms, both of which revolve around the ability of AMPK to inhibit the serine/threonine protein kinase mammalian target of rapamycin (mTOR) (31, 39). Firstly, inhibition of mTOR has been shown to significantly reduce Glut1 transporter activity (40). Given the importance of extracellular-derived glucose in NET formation (21), reduced glucose uptake, secondary to impaired mTOR and Glut1 activity, could contribute to the reduction in NET production and lactate generation that we observed for neutrophils pre-treated with mtDAMPs. Furthermore, this mechanism could contribute to the mtDAMP-induced impairment we reported in neutrophil transmigration since chemotaxis also utilizes extracellular sources of glucose (36). Alongside Glut1, mTOR signaling has been implicated in regulating the expression/activity of hypoxia-inducible factor-1α (HIF-1α) (31). A transcription factor involved in promoting aerobic glycolysis, activation of HIF-1α precedes both NET formation (41) and myeloid cell migration (42). Thus, reduced activity/expression of HIF-1α, secondary to AMPK-mediated inhibition of mTOR, could be an additional/alternative explanation for the mtDAMP-induced reduction in lactate generation, NET generation and neutrophil chemotaxis. Importantly, both these proposed mechanisms could occur in neutrophils without affecting their capacity for ROS production, which we found was significantly increased following mtDAMP treatment. Indeed, as the energy required for ROS generation is derived from endogenous sources of glucose (21), this anti-microbial function could occur in the background of an AMPK driven reduction in extracellular glucose uptake. Our finding of increased ROS production to PMA stimulation for neutrophils pre-treated with mtDAMPs contradicts the post-injury impairment we reported in ROS generation for trauma patients that presented with significantly elevated serum concentrations of mtDAMPs. We believe this discrepancy may be attributable to trauma-induced changes in the composition of the circulating neutrophil pool. For example, traumatic injury results in the emergence into circulation of IGs and CD16^BRIGHT^ CD62L^DIM^ neutrophils, both of which exhibit impaired stimulus-induced ROS generation (43, 44). In contrast, healthy subjects, who served as the cohort for our mtDAMP pre-treatment experiments, possess a homogenous pool of fully-functional mature neutrophils that would exhibit a greater capacity to respond to stimulation.

Although we have shown that neutrophils pre-treated with compound C exhibit increased NET generation upon PMA stimulation, inhibition of AMPK only partially ameliorated the reduction in NET formation that occurred with mtDAMP treatment. Other factors aside from AMPK activation must therefore be involved in mediating the mtDAMP-induced suppression of NET production. Recently, through a proposed mechanism of action that involved the prevention of membrane rupture, lactoferrin, an iron-binding glycoprotein stored within the secondary granules of neutrophils, was found to suppress NET release triggered by PMA stimulation (45). Indicative of a defect in the latter stages of NET production, we showed chromatin decondensation in the absence of DNA release was a feature of mtDAMP-treated neutrophils. Thus, based on this observation and the fact that exposure to mtDAMPs promotes neutrophil degranulation (23, 24, 46), we suggest that a mtDAMP-induced release of lactoferrin could represent an additional mechanistic explanation for the impairment in NET formation that occurs following mtDAMP exposure.

This study has some limitations. Conducted at a single major trauma center, the results of our prospective observational study are based on the analysis of a small number of patient samples, meaning our findings require validation in larger independent cohorts. This point is particularly pertinent to our analysis of pre-hospital blood samples, where inter-individual variability in immune cell number and volume of blood collected at the scene of injury meant we were unable to perform all assays on each patient at this time point. As a heterogeneous collection of proteins, lipids and DNA, no study to date has quantified the exact concentration of mtDAMPs released into the circulation post-injury. Thus, our *in vitro* treatment of neutrophils with 40 or 100 μg/ml mtDAMPs may not be physiologically relevant. However, these doses match those used in previous *in vitro* based studies that have examined the effect of mtDAMP exposure on neutrophil anti-microbial function (23, 24, 46). Similarly, our decision to use the phorbol ester PMA as an agonist may be considered a study limitation. However, as a potent stimulus, it allowed us to study maximal neutrophil responses. Moreover, as the agonist of choice for all previous trauma-based studies that had examined stimulus-induced NET production post-injury (1, 10, 13), our use of PMA enabled us to compare our observations to those in the published literature.

In summary, this is the first study to describe a quantitative post-trauma reduction in NET formation as an immediate on-scene phenomenon, which is accompanied by aberrant intracellular signaling and cell metabolism. In addition, we have shown that the post-injury reduction in NET generation can be recreated *in vitro* by treating neutrophils isolated from healthy subjects with mtDAMPs prior to PMA stimulation. Thus, our data support the suggestion that the release of mtDAMPs from damaged tissue is a contributory factor in the reduction in neutrophil function that occurs post-injury (1, 3, 9–11).

## Data Availability

The datasets generated for this study are available on request to the corresponding author.

## Author Contributions

JH designed the study, performed experimental work, analyzed data, and wrote the manuscript. RD performed experimental work and analyzed data. PH critically appraised the manuscript and JL conceptualized the study and contributed to writing the manuscript.

### Conflict of Interest Statement

The authors declare that the research was conducted in the absence of any commercial or financial relationships that could be construed as a potential conflict of interest.
