# Supplementary material for: Traumatic Injury and Exposure to Mitochondrial-Derived Damage Associated Molecular Patterns Suppresses Neutrophil Extracellular Trap Formation
Source: Front Immunol. 2019 Apr 2;10:685. doi: 10.3389/fimmu.2019.00685 (PMC6455291; doi:10.3389/fimmu.2019.00685)
Supplement: Supplementary file 1 [file Data_Sheet_1.docx]

**
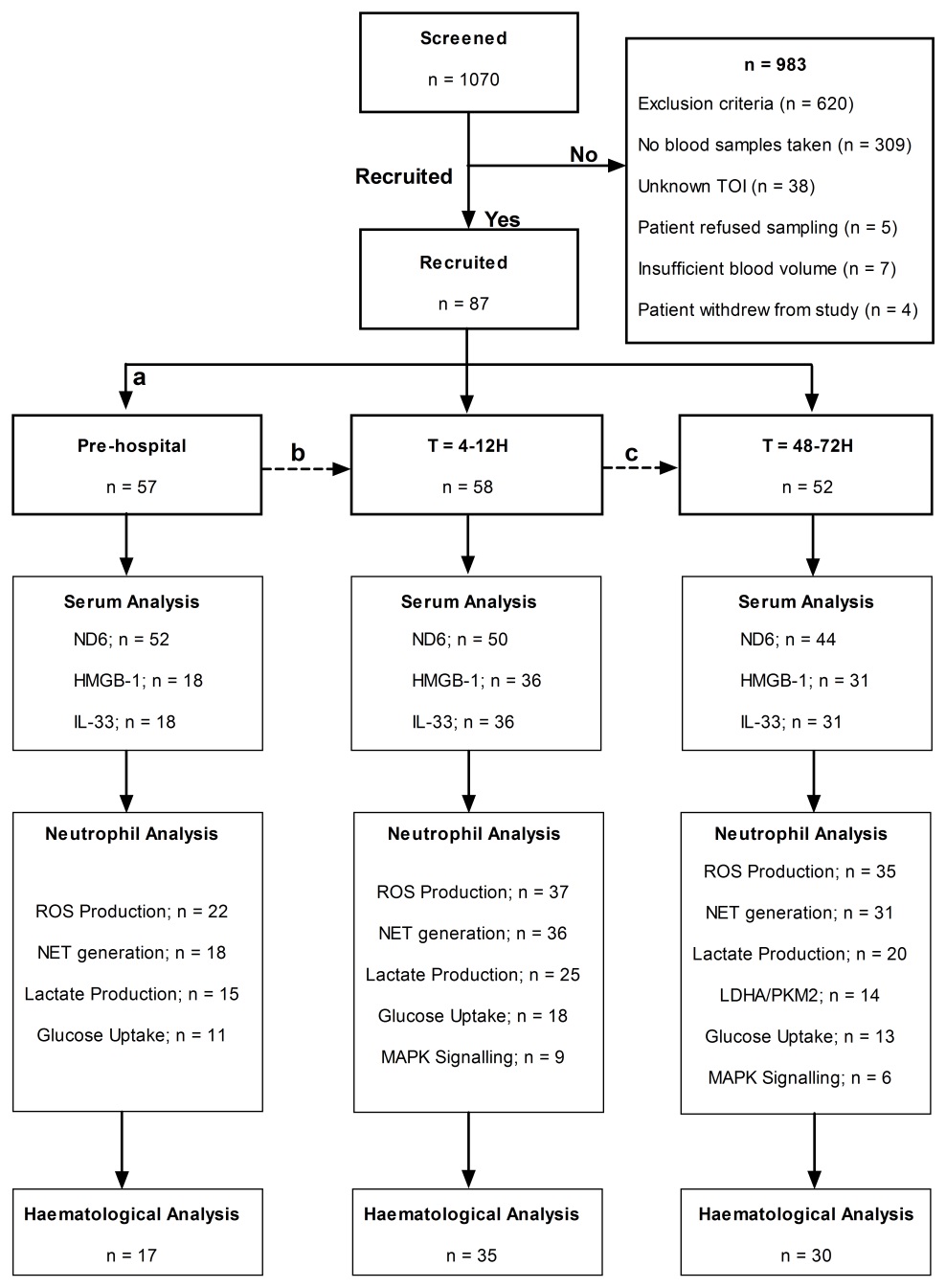
**

**Supplementary Figure 1. CONSORT diagram showing recruitment and analysis of study subjects.** ^a^Insufficient cell recovery from pre-hospital blood samples and equipment breakdown explains the difference in patient number between recruitment (n=87) and analysis of immune function for pre-hospital bloods (n=57). ^b^Due to mortality (n=1), difficulty in bleeding (n=1) and refusal of sampling (n=1), blood samples were not obtained from 3 subjects at the 4-12H time-point for whom analysis of pre-hospital samples had been conducted. Blood samples were however obtained from 4 patients whose pre-hospital bloods were not analysed due to small volume (n=3) or equipment breakdown (n=1). ^c^Between the 4-12H and 48-72H timepoints, samples were not acquired from 8 patients due to difficulty in bleeding (n=4), refusal of sampling (n=1), small blood volume (n=1) or hospital discharge (n=2). However, blood samples were acquired 48-72H post-injury from 1 patient who was difficult to bleed at the 4-12H timepoint and from 1 patient from whom small blood volumes were collected at the pre-hospital and 4-12H time points. Insufficient sample volume, low cell recovery and equipment breakdown accounts for the differences in patient numbers between each parameter analysed. HMGB-1, High mobility group box-1; IL-33, Interleukin-33; LDHA, Lactate Dehydrogenase; MAPK, Mitogen-activated protein kinase; ND6, Mitochondrial encoded NADH dehydrogenase 6; NET, Neutrophil extracellular traps; PKM2, Pyruvate Kinase; ROS, Reactive oxygen species; TOI, Time of injury.

**
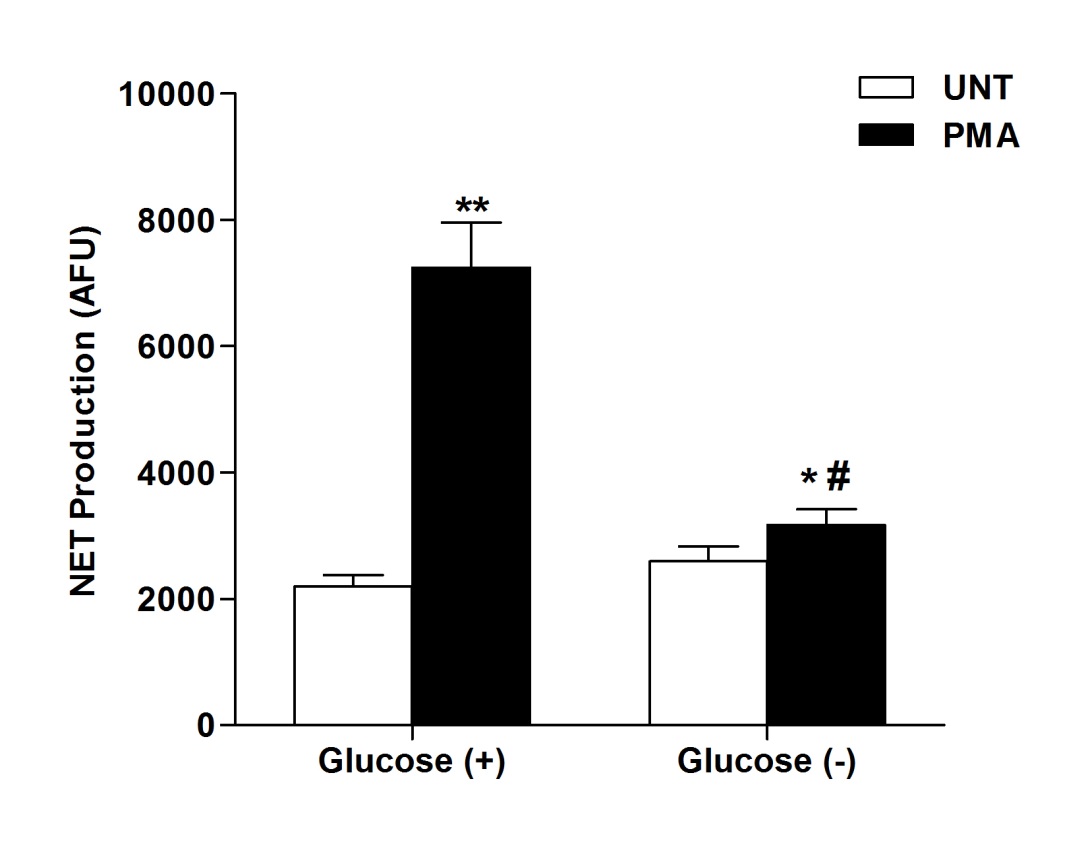
**

**Supplementary Figure 2. Glucose uptake is required for NET generation.** PMA-induced

NET formation by neutrophils cultured in glucose containing or glucose free media. NET

production was measured as DNA concentration in cell-free culture supernatants (n=6).

^*^p<0.01, ^**^p<0.001 vs untreated sample in matched media, ^#^p<0.001 vs. PMA-induced NET

formation in glucose containing media.

**
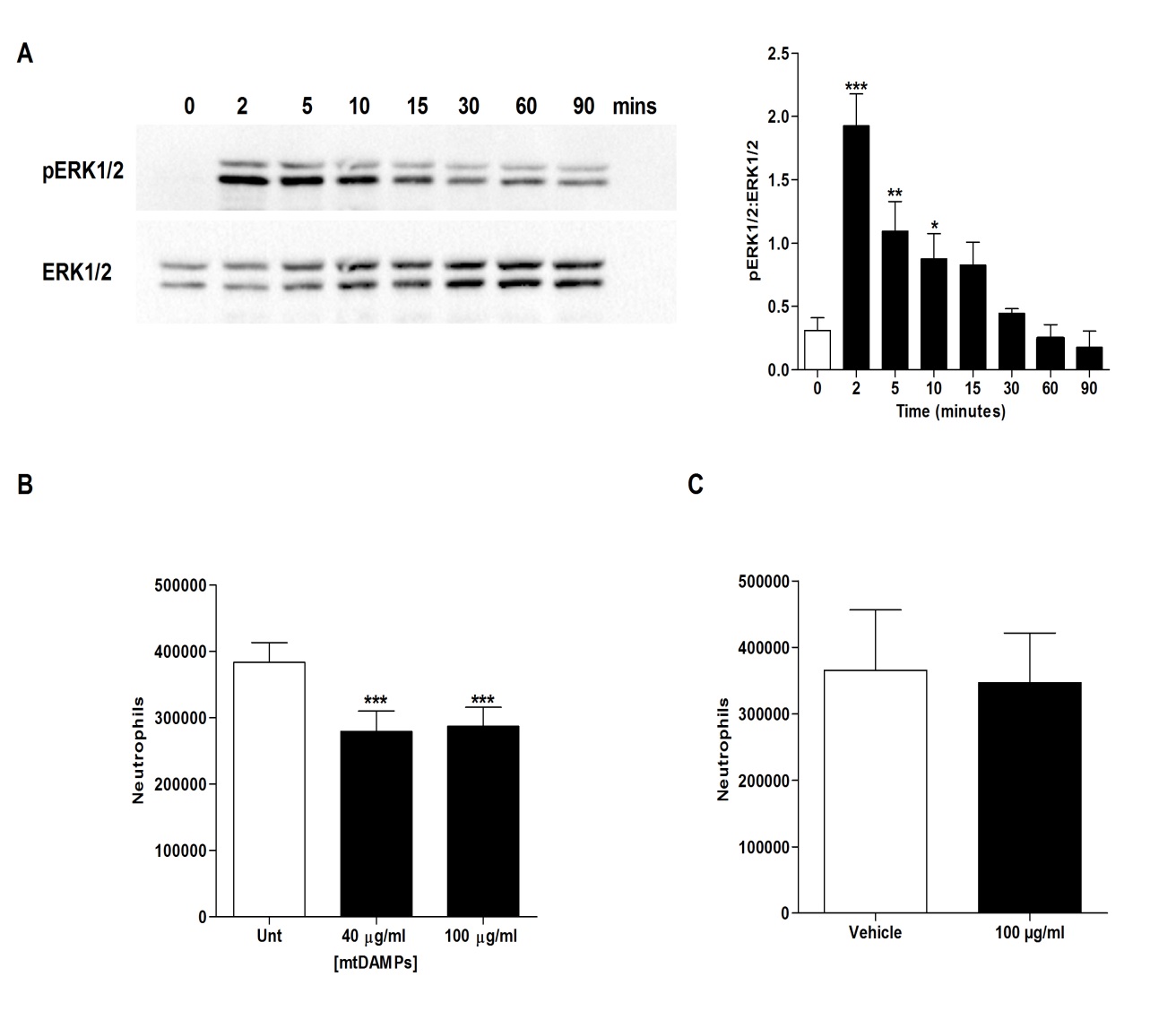
**

**Supplementary Figure 3. MtDAMP stimulation of neutrophils activates ERK 1/2 MAPK signalling and inhibits their migration towards the chemokine LTB_4_. (A)** Activation of the MAPK ERK 1/2 was assessed by Western blotting in freshly isolated neutrophils treated for 2-90 minutes with 100 µg/ml mtDAMPs. Blots are representative of 3 independent experiments, with densitometric data depicted in the accompanying histogram. ^*^p<0.01, ^**^p<0.001, ^***^p<0.0001 vs. 0 minutes. **(B-C)** Neutrophil migration towards 1 nM leukotriene B4 (LTB_4_) was determined following pre-treatment with whole mtDAMP preparations (**B**, n=10) or mtDNA (**C**, n=5). Data are the number of neutrophils that migrated across the transwell in the assay period and are presented as mean±SEM. ^***^p<0.0001 vs. vehicle.

**Supplementary Table 1. Surface phenotype of resting and mtDAMP-treated neutrophils.**

|  | **Untreated** | **MtDAMP-treated**  **(100 µg/ml)** |
| --- | --- | --- |
| **CD62L**  **CD11b**  **CXCR1**  **CXCR2** | 35, 763 ± 2, 057  48, 214 ± 4, 380  25, 400 ± 1, 722  20, 930 ± 2, 285 | 1, 557 ± 119^***^  114, 725 ± 6, 617^***^  22, 316 ± 1, 818^**^  7, 447 ± 868^***^ |

^**^p<0.005, ^***^p<0.0001 versus untreated. Data are presented as mean ± standard error of mean of 10 independent experiments.

**Scans of Full Length Western Blots**

**Blots for Figure 3B**

**
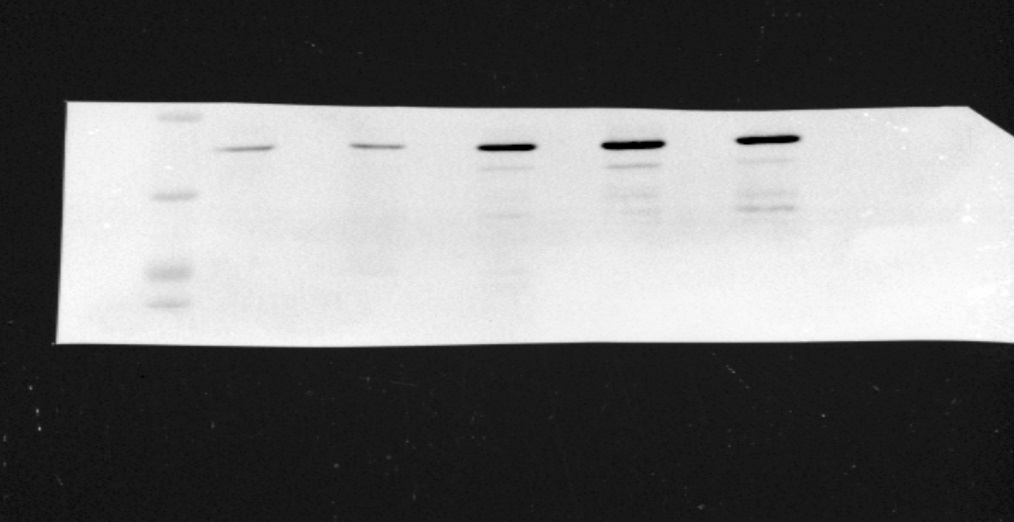
HC Blot**

**pP38**

**
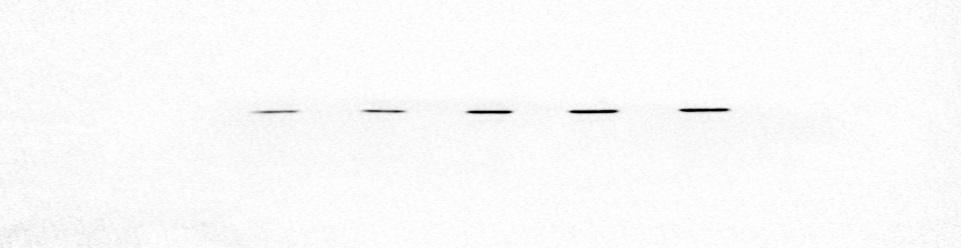
**

**P38**

**Patient 4-12H Blot**

**
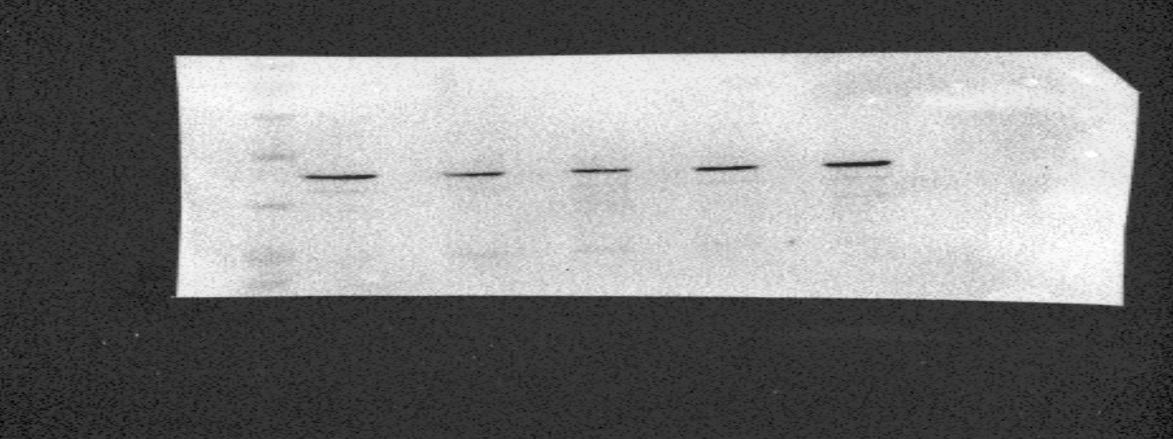
**

**pP38**

**
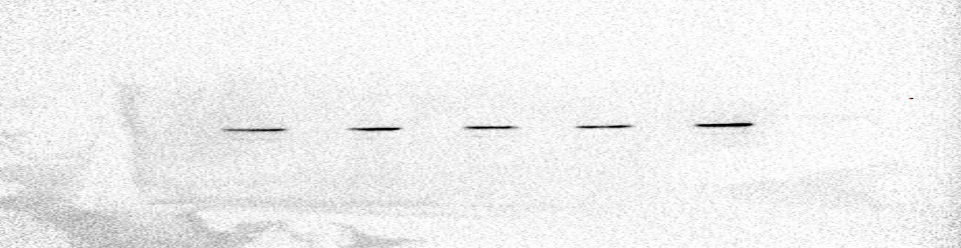
**

**P38**

**Patient 48-72H Blot**

**
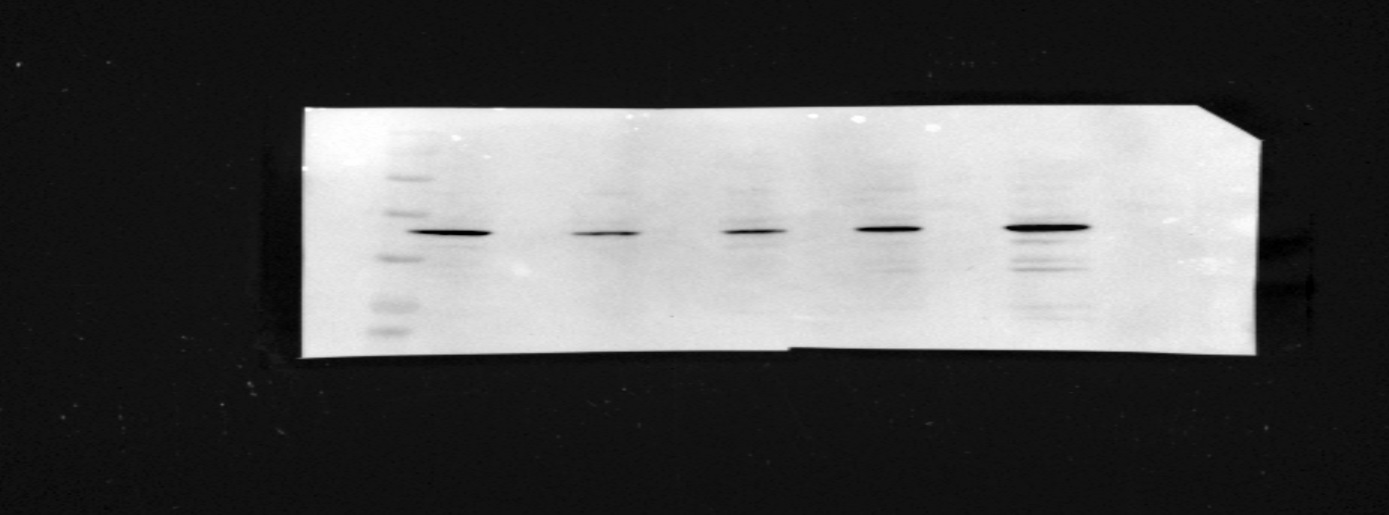
**

**pP38**

**
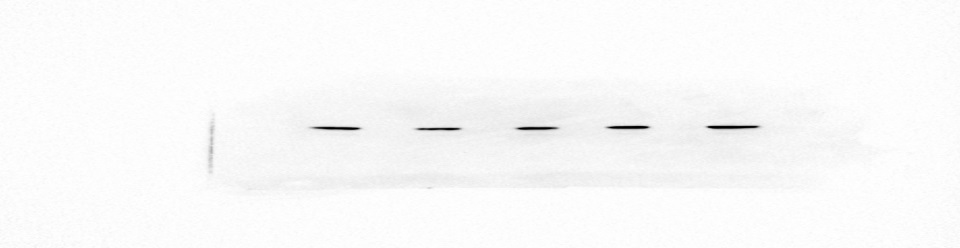
**

**P38**

**Blots for Figure 4B**

**HC Blot**

**
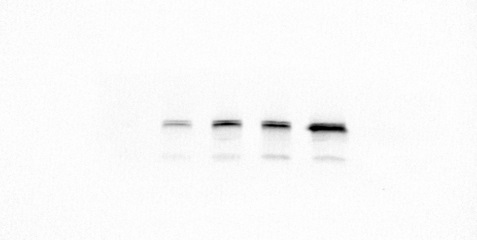
**

**pERK1/2**

**
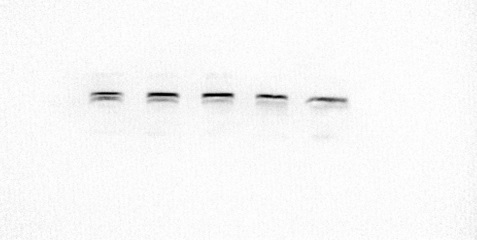
**

**ERK1/2**

**Patient 4-12H Blot**

**
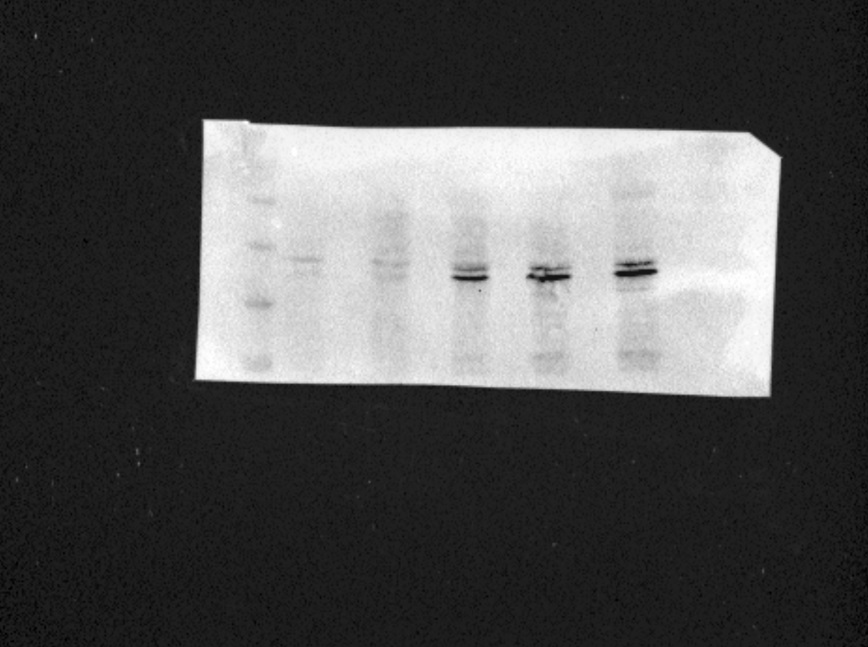
**

**pERK1/2**

**
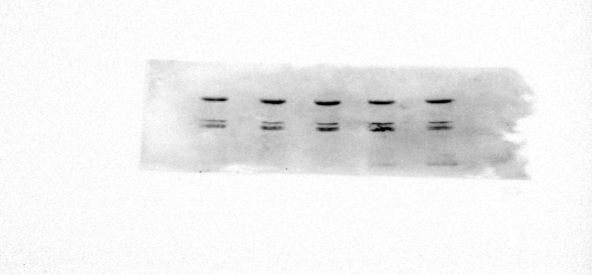
**

**ERK1/2**

**Patient 48-72H Blot**

**
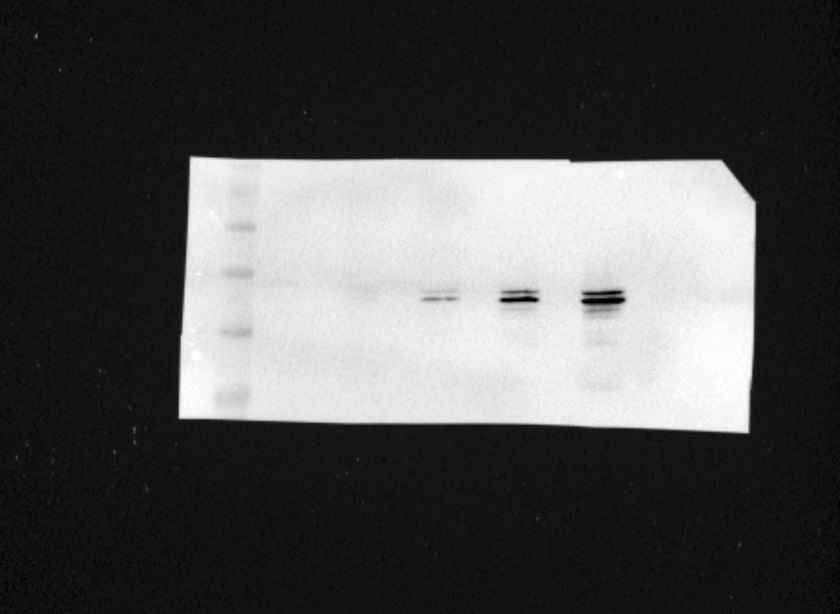
**

**pERK1/2**

**
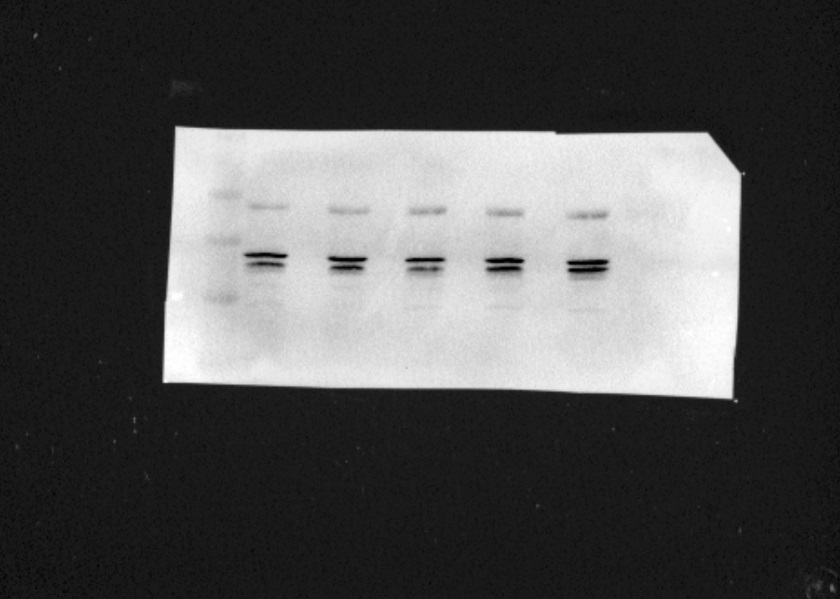
**

**ERK1/2**

**Blots for Figure 5E**

**
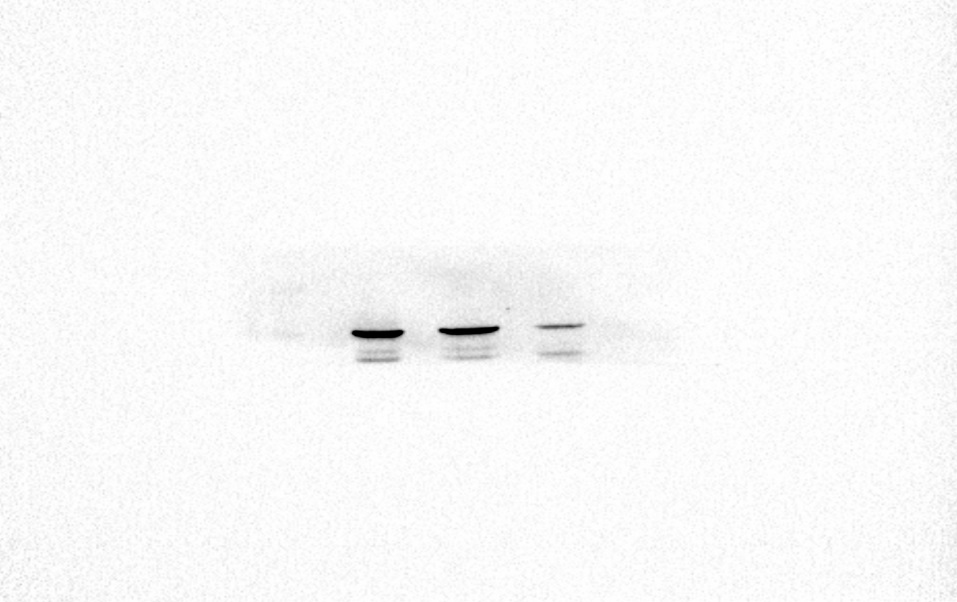
PKM2 Blot**

**LDHA Blot**

**Actin Control Blot**

**
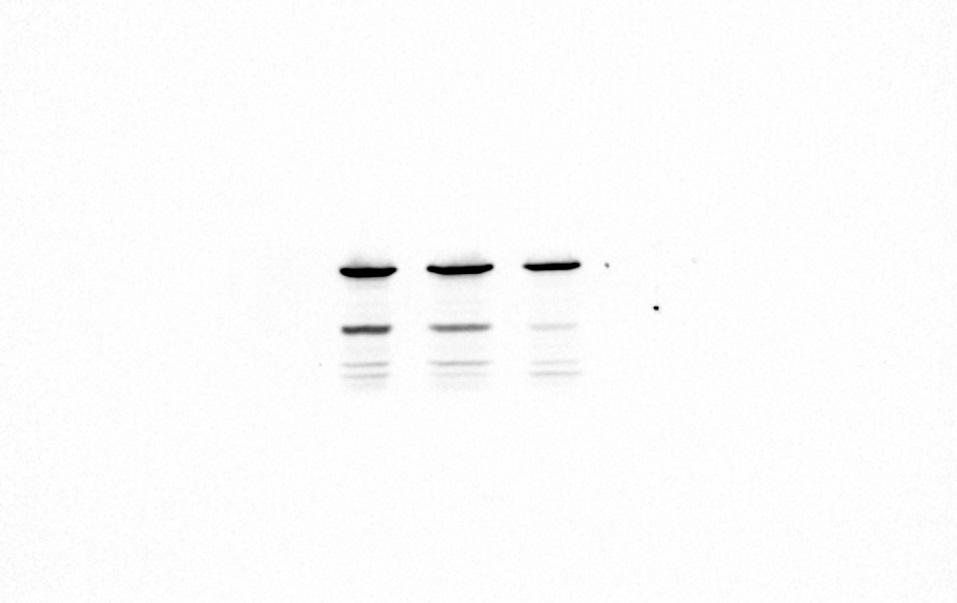
**

**
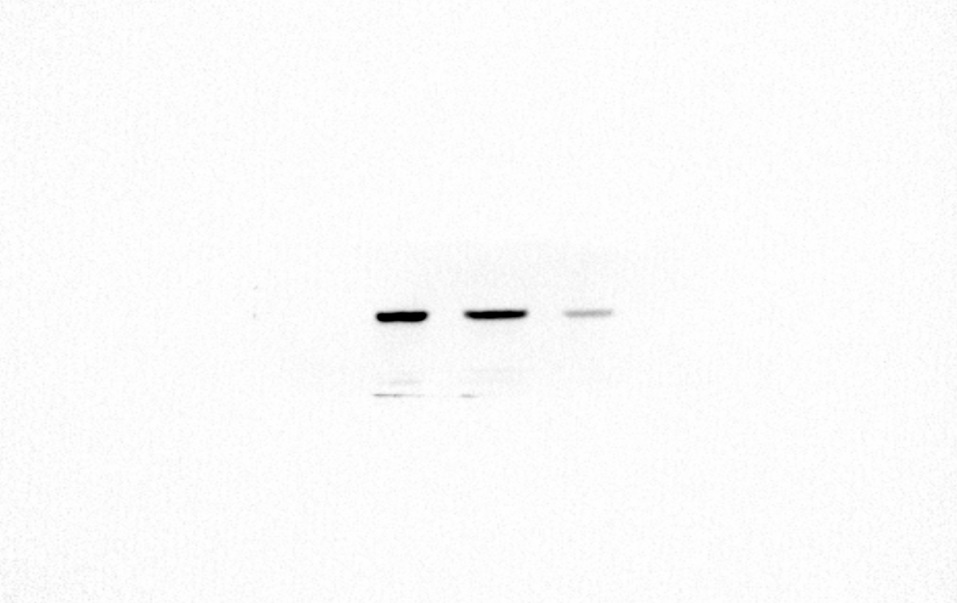
**

**Blots for Figure 7A**

**
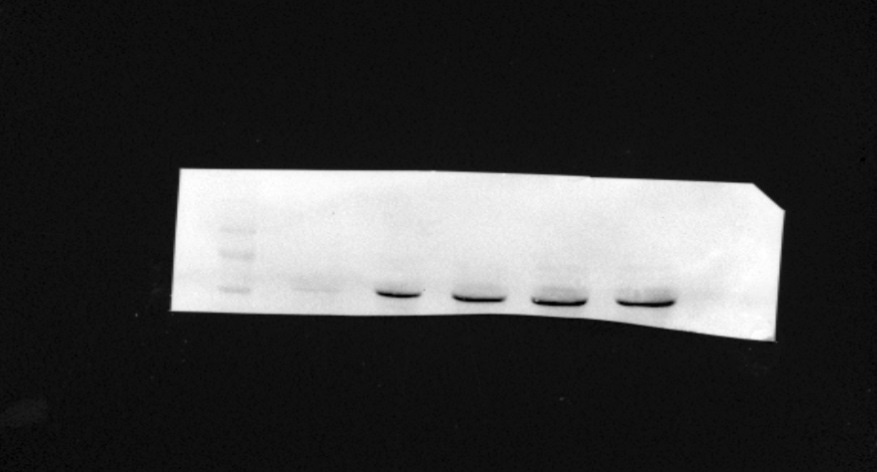
**

**pAMPK**

**
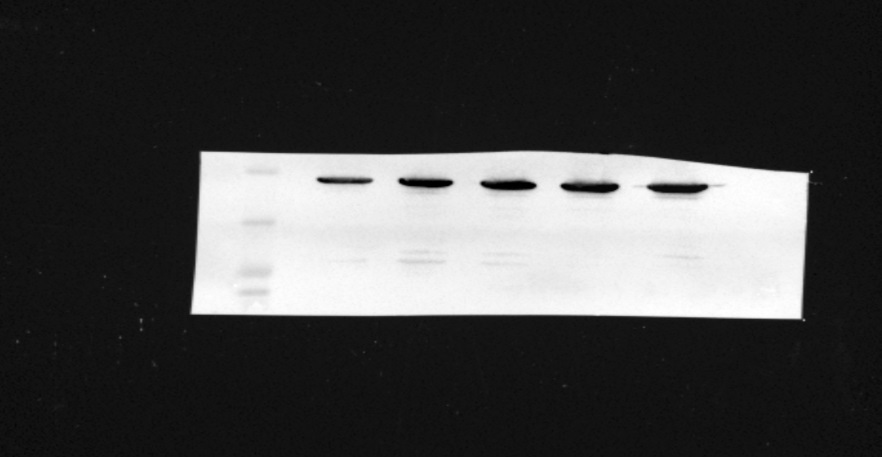
**

**Actin**

**Blots for Figure 7B**

**
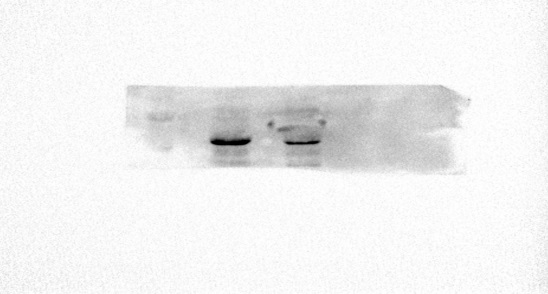
**

**pAMPK**

**
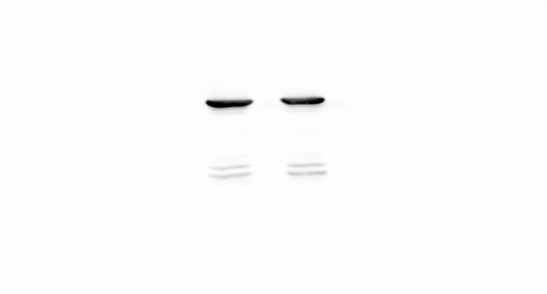
**

**Actin**

**Blots for Figure 7C**

**
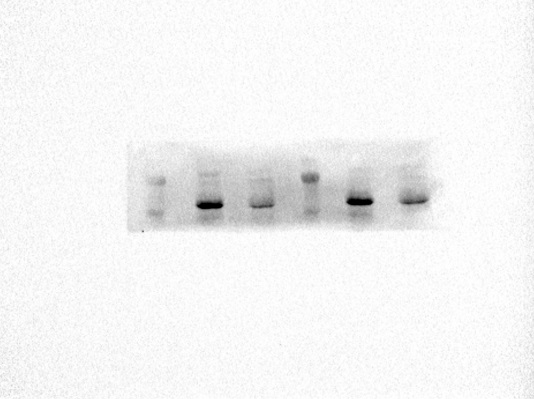
**

**pAMPK**

**
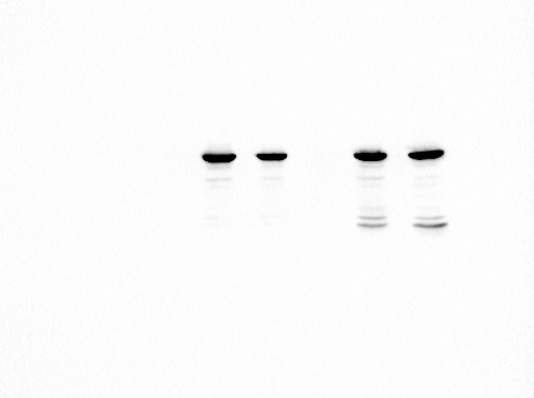
**

**Actin**

**Blots for Supplementary Figure 3**

**
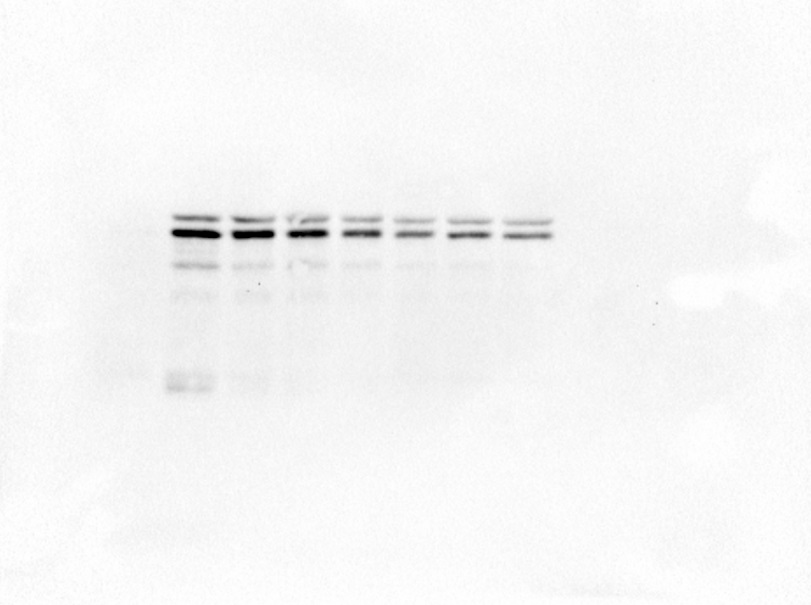
**

**pERK1/2**

**
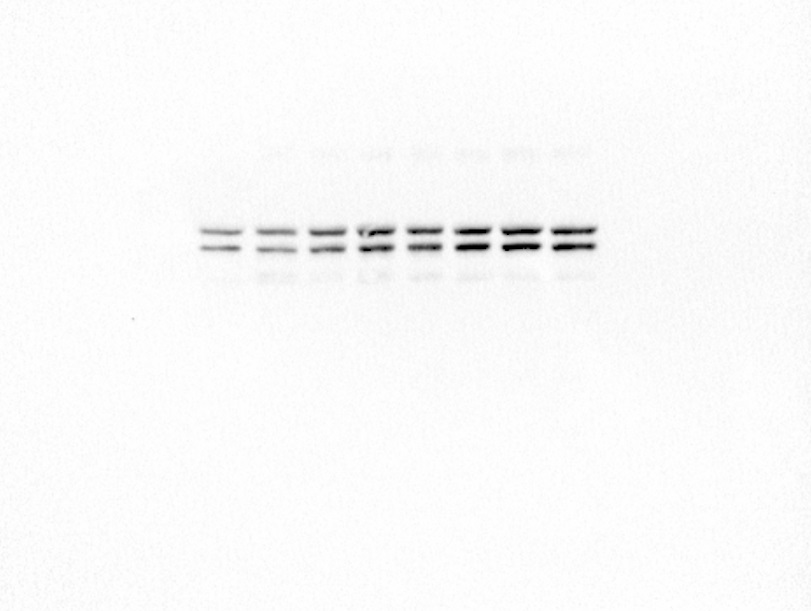
**

**ERK1/2**
